# Scientific Machine Learning for Guided Wave and Surface Acoustic Wave (SAW) Propagation: PgNN, PeNN, PINN, and Neural Operator

**DOI:** 10.3390/s25051401

**Published:** 2025-02-25

**Authors:** Nafisa Mehtaj, Sourav Banerjee

**Affiliations:** Integrated Material Assessment and Predictive Simulation Laboratory (iMAPS), Department of Mechanical Engineering, Molinaroli College of Engineering and Computing, University of South Carolina, Columbia, SC 29201, USA; nmehtaj@email.sc.edu

**Keywords:** scientific machine learning, wave propagation, physics-guided neural network, physics-informed neural network, physics-encoded neural network, neural operator

## Abstract

The governing Partial Differential Equation (PDE) for wave propagation or the wave equation involves multi-scale and multi-dimensional oscillatory phenomena. Wave PDE challenges traditional computational methods due to high computational costs with rigid assumptions. The advent of scientific machine learning (SciML) presents a novel paradigm by embedding physical laws within neural network architectures, enabling efficient and accurate solutions. This study explores the evolution of SciML approaches, focusing on PINNs, and evaluates their application in modeling acoustic, elastic, and guided wave propagation. PINN is a gray-box predictive model that offers the strong predictive capabilities of data-driven models but also adheres to the physical laws. Through theoretical analysis and problem-driven examples, the findings demonstrate that PINNs address key limitations of traditional methods, including discretization errors and computational inefficiencies, while offering robust predictive capabilities. Despite current challenges, such as optimization difficulties and scalability constraints, PINNs hold transformative potential for advancing wave propagation modeling. This comprehensive study underscores the transformative potential of PINN, followed by recommendations on why and how it could advance elastic, acoustic, and guided wave propagation modeling and sets the stage for future research in the field of Structural Health Monitoring (SHM)/Nondestructive Evaluation (NDE).

## 1. Introduction

Partial Differential Equations (PDEs) serve as the fundamental mathematical framework for modeling a wide range of physical phenomena and natural processes [1]. In particular, the wave equation has been successful in capturing a broad spectrum of physics problems from many fields like structural health monitoring (SHM) [2], nondestructive evaluation (NDE) [3], geophysics [4], acoustics [5], medical imaging [6], fluid dynamics [7], electromagnetics [8], and many other fields. Recently, there has been a notable surge in academic interest in wave-based SHM techniques. This interest is primarily driven by the goal of developing automatic continuous monitoring systems that improve structural functionality, reduce maintenance costs, and extend the operational lifespan of structures [9]. Acoustic, elastic, and guided waves are one of the foundational tools of SHM, providing critical insights into structural integrity and material properties [2,10,11,12,13]. Thus, solving these three types of wave equations is extremely important.

Since d’Alembert first introduced the wave equation, significant research has focused on solving it in various forms using different analytical approaches [14]. Some well-established analytical methods include the characteristic curves, variation of parameters, Green’s functions, Fourier and Radon transforms, and many more. However, these methods are generally inadequate for addressing complex geometries and higher-dimensional domains. In many such cases, the analytical solution does not exist either [15,16]. With the introduction of faster computers, numerical methods have evolved as a long-standing tradition for wave propagation modeling over the last 50 years [17]. To name a few of them: Spectral Element Method (SEM) [18,19,20], Finite Element Method (FEM) [21], and Finite Difference Method (FDM) [22], Boundary Element Method (BEM) [23], Mass Spring Lattice Model (MSLM) [24], Finite Strip Method (FSM) [25,26], Peri-elastodynamic [27], Cellular Automata [28,29], Elastodynamic Finite Integration Technique (EFIT) [30], etc. These techniques are highly sophisticated and have been serving effectively for decades. Nevertheless, these methods also have certain drawbacks. A key concern is that they become computationally very demanding due to their mesh-based nature when tackling higher dimensional problems. This issue is called the Curse of Dimensionality (CoD) [31]. Another common problem is discretization error. The discretization error occurs when the grid size is not sufficiently small compared to the wavelength to capture the desired resolution. Similarly, to capture the temporal evolution effectively, the sampling size needs to be small enough to accommodate the constraints imposed by the meshing [32]. In addition, the Gibbs phenomenon is a well-known numerical artifact characterized by spurious oscillations near non-smooth or discontinuous regions. This issue is frequent in most computational methods due to the reliance on polynomials, piecewise polynomials, and other basis functions [33,34,35]. All of these methods have their own advantages and disadvantages, including the common issue of high computational cost [36].

As an effort to reduce the computational burden, scholars in this field proposed several semi-analytical methods. Among them, the Distributed Point Source Method (DPSM) stands out due to its use of displacement and stress Green’s function in its meshless semi-analytical problem formulation [37]. It avoids inherent spurious reflection, making it more accurate than FEM. Also, the frequency domain DPSM is much faster than FEM, BEM, and SEM [37]. This model, however, tends to match the required conditions at some specific points (apex), which makes the simulated wavefield a bit weaker than expected [38]. Another notable time domain method, similar to EFIT, is the Local Interaction Simulation Approach (LISA) [39,40,41]. It is an expensive computational model as it requires additional local interaction of material points in temporal and spatial domains. Parallel computing is needed to simulate this method efficiently. High computational demands make the existing numerical and semi-analytical models impractical for real-world applications [42].

Though the list of non-exhaustive challenges of all these physics-driven methods may seem like a barrier, they play a crucial role in making the field data-rich. The exponential growth of data opens a new door for researchers to shift from computationally heavy to efficient and low-cost data-driven modeling of the wavefield. At this point, Machine Learning (ML) becomes a game changer. Until now, there have been many attempts to simulate wave propagation using traditional data-driven ML models [43,44,45,46]. Scientific Machine Learning (SciML), a burgeoning and integrative subset of ML, has emerged as a revolutionary research frontier to address engineering problems, specifically. What sets SciML apart from traditional ML is its integration of scientific domain knowledge into the process. The term “domain knowledge” refers to physical principles, constraints, computational simulations, correlations in space and time, etc. Therefore, while the physics-driven models set the stage by providing the ground truth, SciML can now efficiently solve intricate forward and inverse engineering problems [47,48].

Existing literature highlights the exceptional capability of SciML to address several challenges associated with traditional physics-based methods effectively. These models offer notable speedup upon successful training, substantially cutting down on computational time [49]. The use of automatic differentiation [50] for gradient calculation eliminates the need for discretization, effectively addressing the issue of CoD. Advanced algorithms such as Convolutional Neural Network (CNN), Recurrent Neural Network (RNN), Physics-informed Neural Network (PINN), and many others can efficiently solve the 3D wave equations [51,52,53]. Instead of employing linear piecewise polynomials, a non-linear representation of neural network (NN) is used to capture the non-linearity of the PDE solution. In traditional models, “the finer the mesh, the greater the accuracy”, is a major reason for the growing computational burden. In contrast, the performance of SciML models depends on optimized weights and biases of the NN. In this case, the optimization is carried out using gradient-based optimizers [54] instead of linear solvers [55,56]. The primary challenge in SciML lies in determining which domain knowledge should be leveraged for modeling and how to effectively integrate this knowledge into the computational framework. Researchers are actively advancing this branch, resulting in the development of numerous innovative modeling approaches such as “physics-informed”, “physics-constrained”, “physics-enabled”, “physics-based”, “physics-guided”, and “theory-guided” in a very short period. The literature currently lacks precise nomenclature for these models [57,58]. An excellent review by Faroughi et al. [59] on scientific computing shed light on the classification of the different SciML methods. The research group categorized the SciML models into four prime approaches: Physics-guided Neural Network (PgNN), Physics-informed Neural Network (PINN), Physics-enabled Neural Network (PeNN), and Neural Operators (NOs).

**Figure 1:**
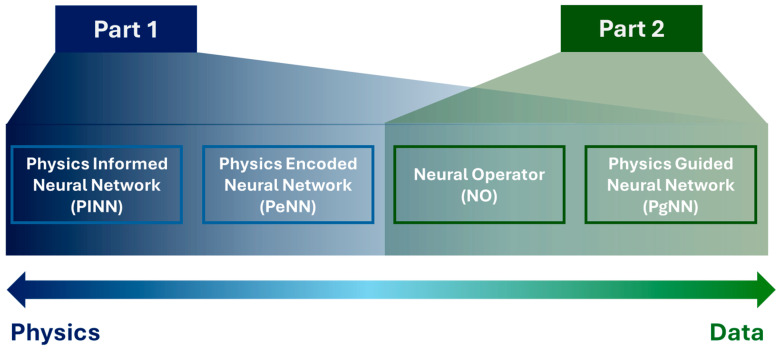
Outline of the two-fold study for the application of physics-driven artificial intelligence tailored for wave propagation.

The primary focus of this article is to explore existing literature that integrates these four SciML models with the known physics of wave propagation, providing deeper theoretical insights into algorithms. The study presents a comprehensive review of acoustic, elastic, and guided wave modeling using the SciML models in the field of Structural Health Monitoring (SHM)/Nondestructive Evaluation (NDE). However, the investigation highlights that research in this area is currently limited in scope. Thus, the discussion has been extended to the studies incorporating SciML into wave equation-based imaging techniques to bridge the knowledge gaps and expand future research directions. To summarize the contribution, this article answers four critical questions: (i) how SciML algorithms have evolved in the context of physics infusion, (ii) what has been done in the SHM sectors, (iii) what has been done in the other relevant sectors, and (iv) what more can be done. The article is divided into two parts. The first part addresses physics-intensive SciML methods, which are PINNs and PeNNs. The second part focuses on data-driven SciML approaches, including PgNNs and NOs. Figure 1 clearly depicts the topics covered in this two-part review. As there is barely any work on wave equation modeling using the PeNN model, the article confines its scope to only presenting the model’s algorithm. Therefore, Part 1 solely concentrates on studies involving PINN to simulate the wavefield. The remainder of this article is as follows. Section 2 outlines the underlying physics of acoustic, elastic, and guided waves. Later, Section 3 covers the evolution of the four SciML models and their architecture in the context of the different ML modeling approaches. Section 4 reviews the studies on PINN applications for modeling acoustic, elastic, and guided waves. Finally, Section 5 concludes the study with potential achievements, future challenges, and probable solutions in this field.

## 2. Fundamentals of Elastic, Acoustic, and Guided Wave

In this article four SciML methods primarily, PINN, PeNN, PgNN, and NO are discussed in the context of wave propagation while solving partial differential equations particularly associated with wave propagation. Wave in solid and fluid media propagates as a stress or pressure wave (For example, stress wave propagates through the earth crust after an earthquake) abiding by certain physical laws fundamentally linked to the Euler–Lagrange equation derived from the Hamilton principle. The equation is applicable to each point in the media. It is a partial differential equation that describes how an energy perturbation or transmission should propagate into the media while all the conservation principles (mass, energy, linear and angular momentum, Noether current) and geometric principle (strain-displacement relation and compatibility conditions) are simultaneously satisfied. Hence, it is necessary to understand such partial differential equations in fluid and solid media. This section discusses the fundamentals of elastic, acoustic, and guided wave propagation in the context of ultrasonic NDE and SHM where ultrasonic transducers are actively used to transmit energy. A typical ultrasonic NDE setup always concerns fluid and solid media with fluid–solid interfaces. In the following sections, the generalized Einstein index notation in three dimensions is used, if not explicitly mentioned. From the fundamentals of classical mechanics applying Hamiltonian principles on the Lagrangian density of a system, Navier’s equation of motion is derived as

(1) $$\sigma_{ij,j}+f_{i}=\rho \ddot{u_{i}},$$

where $\sigma_{ij}$ is the stress tensor of the media, ρ is density of the media, and $f=f_{i}{\hat{e}}_{i}$ is body force.

### 2.1. Acoustic Wave Equation

When ultrasonic energy is transmitted using a transducer in water or in any incompressible fluid media, the energy propagates as a pressure wave in all possible directions, resulting in spherical wave fronts. The wave fronts in fluid propagate with a constant velocity irrespective of the frequency of the wave actuated in the media, make them nondispersive, i.e., angular frequency (ω) vs. wave number (k) relationship is linear with a constant slope. Perfect incompressible fluid cannot support any shear stresses, and pressure at a point is equal and compressive from all possible directions. This gives us a relation that the normal stresses $\sigma_{11}=\sigma_{22}=\sigma_{33}=-p$, where $p\left(x,t\right)$ is pressure at a point at an instance in a fluid media. The negative sign is to designate compressive stress that always prevails in fluid. Gradient of pressure ∇p is force and, imposing Hamiltonian principles or Newton’s second law, force balance equation in fluid can be written as

(2) $$-\nabla p+f=\rho \ddot{u}\;or\;\langle {-p}_{,i}+f_{i}=\rho {\ddot{u}}_{i}\rangle {\hat{e}}_{i}\;or\;simply\;{-p}_{,i}+f_{i}=\rho {\ddot{u}}_{i}$$

where f is body force; u is the acoustic displacement field; i is used to represent index notation taking values 1, 2, and 3; ρ is density of the fluid. To find how the radius of the vector front changes over time divergence, i.e., taking a dot product with the gradient, vector ∇ of the force field would read

(3) $$-\nabla \;\cdot \nabla p+\nabla \cdot f=\rho \nabla \cdot \ddot{u}\;\;\;\;\;\;or\;\;\;\;\;{-p}_{,ii}+f_{i,i}=\rho {\ddot{u}}_{i,i}$$

Assuming there is an isotropic constitutive relation for fluid with bulk modulus (K), and that summation of normal strains becomes $e_{kk}$, the stress would take a form as $\sigma_{ij}=K\delta_{ij}e_{kk}$. Thus, the pressure field would read as

(4) $$-p=L\left(e_{11}+e_{22}+e_{33}\right)=K\left(u_{1,1}+u_{2,2}+u_{3,3}\right)=Ku_{i,i}=\;K\;\nabla \cdot u$$

Substituting Equation (3) into Equation (2), the acoustic wave equation (Navier’s equation) in Equation (1) is obtained as

(5) $${-p}_{,ii}-\frac{\rho }{K}\ddot{p}+f_{i,i}=0\;\;\;\;\;\;\;\;or\;\;\;{-p}_{,ii}-\frac{1}{c_{f}^{2}}\ddot{p}+\;f_{i,i}=0$$

where $c_{f}=\sqrt{K/\rho }$ is the acoustic wave velocity in the fluid media, and $\ddot{p}$ is the second order derives of pressure with respect to time. Further neglecting body force, the final form of wave equation in fluid would result in

(6) $$\nabla^{2}p-\frac{1}{c_{f}^{2}}\;\frac{\partial^{2}p}{\partial t^{2}}=0\;\;\;\;\;\;\;\;\;or\;\;\;\;\;\;\left(\frac{\partial^{2}p}{\partial {x_{1}}^{2}}+\frac{\partial^{2}p}{\partial {x_{2}}^{2}}+\frac{\partial^{2}p}{\partial {x_{3}}^{2}}\right)=\frac{1}{c_{f}^{2}}\;\frac{\partial^{2}p}{\partial t^{2}}\;$$

where $\nabla^{2}=\nabla \cdot \nabla$ signifies the divergence of the gradient of a scalar field.

### 2.2. Elastic Wave and Guided Wave Equation

The stress tensor for a generalized linear, solid media could be written as

(7) $$\sigma_{ij}=C_{ijkl}(x_{m})e_{kl}$$

where $C_{ijkl}$ is the constitutive matrix at a spatial coordinate $x_{m}$, and $e_{kl}$ is the strain vector. If the material is isotropic, the stress would read

(8) $$\sigma_{ij}=L\delta_{ij}e_{kk}+2me_{ij}$$

where L and m are two Lamé constants of isotropic material, $\delta_{ij}$ is the Kronecker delta function, strain tensor $e_{kl}=\frac{1}{2}\left(u_{k,l}+u_{l,k}\right)$, and $u=u_{i}{\hat{e}}_{i}$ is the elastic displacement field. Substituting the strain-displacement relation in Equations (7) and (8), and substituting the stress expression in Equation (1), the elastic wave equation in general anisotropic and isotropic solids are derived as follows, respectively, in Equations (9) and (10)

(9) $$C_{ijml}\frac{\partial^{2}u_{m}}{\partial x_{j}\partial x_{l}}+f_{i}=\rho {\ddot{u}}_{i}$$

(10) $$\left(L+2m\right)u_{j,ji}-m\epsilon_{ijk}\epsilon_{kpq}u_{q,pj}+f_{i}=\rho {\ddot{u}}_{i}$$

where $\epsilon_{ijk}$ is a Levi-Civita symbol, commonly known as a permutation symbol. These equations could be numerically solved as time-domain problems with direct computational methods. The geometry of the problem will define if the solution results in an elastic bulk wave solution or in a guided wave solution. However, some analytical understanding is still required to verify the results. A few analytical understandings are reviewed below.

Applying Helmholtz decomposition u=∇ϕ+∇×ψ in isotropic solid, two elastic wave equations emerged where ϕ is a scalar wave potential signifies longitudinal wave (P-wave) and ψ is the vector wave potential, with three components signifying shear waves (S-wave). These equations are for longitudinal waves, in Equation (11), and shear waves, in Equation (12).

(11) $$\nabla^{2}\phi -\frac{1}{c_{p}^{2}}\ddot{\phi }=0\;\;\;\mathrm{or}\;\;\;\;\left(\frac{\partial^{2}\phi }{\partial {x_{1}}^{2}}+\frac{\partial^{2}\phi }{\partial {x_{2}}^{2}}+\frac{\partial^{2}\phi }{\partial {x_{3}}^{2}}\right)=\frac{1}{c_{p}^{2}}\;\frac{\partial^{2}\phi }{\partial t^{2}}\;$$

(12) $$\nabla^{2}\psi -\frac{1}{c_{s}^{2}}\ddot{\psi }=0\;\;\mathrm{or}\;\;\;\;\left(\frac{\partial^{2}\psi_{i}}{\partial {x_{1}}^{2}}+\frac{\partial^{2}\psi_{i}}{\partial {x_{2}}^{2}}+\frac{\partial^{2}\psi_{i}}{\partial {x_{3}}^{2}}\right)=\frac{1}{c_{s}^{2}}\;\frac{\partial^{2}\psi_{i}}{\partial t^{2}}$$

where $c_{p}=\sqrt{\frac{\left(L+2m\right)}{\rho }}$ and $c_{s}=\sqrt{\frac{m}{\rho }}$ are the P-wave or longitudinal and S-wave or shear wave velocities in the isotropic solid media. Solving the longitudinal(transverse) potentials with suitable, respective initial conditions $\phi (\psi )(x_{j},t=0)=g(G)(x_{j})$ and boundary conditions $\phi (\psi )(x_{j}=0,t)=b(B)(t)$, and substituting the potential solutions into the displacement functions, wave fields in isotropic media could be solved. Usually, monochromatic potential functions at a frequency ω is assumed for the potential

(13) $$\phi =Ae^{i\left(k_{m}^{p}x_{m}\right)}e^{-i\omega t}\;\;\;\;\;\;and\;\;\;\;\;\;\;\;\psi =Be^{i\left(k_{m}^{s}x_{m}\right)}e^{-i\omega t}$$

where $k_{m}^{p}$ and $k_{m}^{s}$ are the P and S wave numbers, respectively, along the direction of propagation $x_{m}$. A and B in Equation (12) are the amplitude of the scalar and vector potentials, respectively, representing polarization. Thus, if the polarization vectors (A and B) are solved, the elastic wave displacement fields are solved simultaneously.

**Figure 2:**
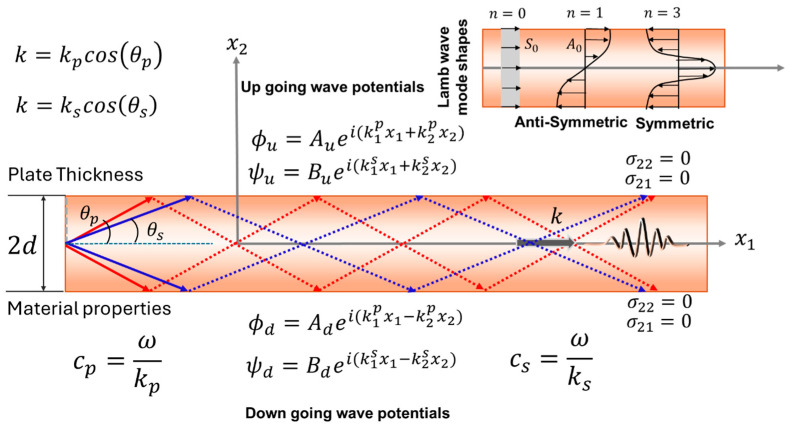
A schematic showing the cross-section of an isotropic wave guide and the relevant wave potential function are used to express the displacement function.

In an isotropic wave guide, to manifest guided wave propagation, the above potentials in Equation (13) must be defined separately for up-going and down-going waves that cause the propagation of the guided waves. Stress-free boundary conditions must be applied on the top and the bottom surfaces of the wave guide. As shown in Figure 2, the four essential potential functions are as follows: $\phi_{u}=A_{u}e^{i\left(k_{1}^{p}x_{1}+k_{2}^{p}x_{2}\right)}$ and $\phi_{d}=A_{d}e^{i\left(k_{1}^{p}x_{1}-k_{2}^{p}x_{2}\right)}$ are up-going and down-going wave potentials, respectively, for totally internally reflecting P-waves. Similarly, $\psi_{u}=B_{u}e^{i\left(k_{1}^{s}x_{1}+k_{2}^{s}x_{2}\right)}$ and $\psi_{d}=B_{d}e^{i\left(k_{1}^{s}x_{1}-k_{2}^{s}x_{2}\right)}$ are the two upgoing and down going vertically polarized S-wave potentials, respectively. Figure 2 shows the wave potential in a graphical sketch for visualization. Here, harmonic temporal term $e^{-i\omega t}$ for a monochromatic wave with frequency ω is omitted. In this case, all other shear potentials are considered to have vanished in the wave guide. Considering the Helmholtz decomposition, and using the fact that all other shear wave potential vanishes, the displacement function along two orthogonal directions can be written as

(14) $$u_{1}=\left[ik_{1}^{p}A_{u}e^{i\left(k_{1}^{p}x_{1}+k_{2}^{p}x_{2}\right)}+ik_{1}^{p}A_{d}e^{i\left(k_{1}^{p}x_{1}-k_{2}^{p}x_{2}\right)}+ik_{2}^{s}B_{u}e^{i\left(k_{1}^{s}x_{1}+k_{2}^{s}x_{2}\right)}-ik_{2}^{s}B_{d}e^{i\left(k_{1}^{s}x_{1}-k_{2}^{s}x_{2}\right)}\right]e^{-i\omega t}$$

(15) $$u_{2}=\left[ik_{2}^{p}A_{u}e^{i\left(k_{1}^{p}x_{1}+k_{2}^{p}x_{2}\right)}-ik_{2}^{p}A_{d}e^{i\left(k_{1}^{p}x_{1}-k_{2}^{p}x_{2}\right)}-ik_{1}^{s}B_{u}e^{i\left(k_{1}^{s}x_{1}+k_{2}^{s}x_{2}\right)}-ik_{1}^{s}B_{d}e^{i\left(k_{1}^{s}x_{1}-k_{2}^{s}x_{2}\right)}\right]e^{-i\omega t}$$

By applying the boundary conditions, the eigen values or the wave modes are solved. Understanding these wave modes is crucial to verifying and validating the numerical or SciML solution results. Solution methods for elastic waves in anisotropic solids, however, are different. Elastic modes in anisotropic media are coupled, where ϕ and ψ potential cannot be uniquely separated.

To solve the wave propagation in anisotropic media, Buchwald decomposition [60] is used with potential functions $\Theta_{1}(x_{j})e^{-i\omega t}$, $\Theta_{2}(x_{j})e^{-i\omega t}$, and $\Theta_{3}(x_{j})e^{-i\omega t}$ where $\Theta_{i}(x_{j})$ are material-dependent spatial functions to be found through boundary conditions. Utilizing Buchwald potential functions, the displacement field in anisotropic solids can be written as

(16) $$u_{1}=\left(\frac{\partial \Theta_{1}}{\partial x_{1}}\right)e^{-i\omega t};\;u_{2}=\left(\frac{\partial \Theta_{2}}{\partial x_{2}}+\frac{\partial \Theta_{3}}{\partial x_{3}}\right)e^{-i\omega t};\;u_{3}=\left(\frac{\partial \Theta_{2}}{\partial x_{3}}-\frac{\partial \Theta_{3}}{\partial x_{2}}\right)e^{-i\omega t}$$

However, to calculate these displacement functions, the potential functions need to be evaluated which are highly material-dependent. The potential functions in a transversely isotropic media with $x_{1}$ axis of symmetry [61] can be expressed as follows:

(17) $$\left.\begin{array}{c} \Theta_{1}=\left[K_{11}\left(C_{u}^{1}e^{i\xi_{1}x_{3}}+C_{d}^{1}e^{-i\xi_{1}x_{3}}\right)+K_{12}\left(C_{u}^{2}e^{i\xi_{2}x_{3}}+C_{d}^{2}e^{-i\xi_{2}x_{3}}\right)\right]e^{i\left(k_{1}x_{1}+k_{2}x_{2}\right)}\\ \Theta_{2}=\left[K_{21}\left(C_{u}^{1}e^{i\xi_{1}x_{3}}+C_{d}^{1}e^{-i\xi_{1}x_{3}}\right)+K_{22}\left(C_{u}^{2}e^{i\xi_{2}x_{3}}+C_{d}^{2}e^{-i\xi_{2}x_{3}}\right)\right]e^{i\left(k_{1}x_{1}+k_{2}x_{2}\right)}\\ \Theta_{3}=\left[C_{u}^{3}e^{i\xi_{3}x_{3}}+C_{d}^{3}e^{-i\xi_{3}x_{3}}\right]e^{i\left(k_{1}x_{1}+k_{2}x_{2}\right)} \end{array}\right\}$$

where $K_{11}$, $K_{12}$, $K_{21},$ and $K_{22}$, and $\xi_{1}$, $\xi_{2},$ and $\xi_{3}$ can be written as:

$$\begin{array}{c} \alpha =\Lambda_{2}\Lambda_{5}=\frac{C_{22}}{\rho }\;\frac{C_{55}}{\rho },\;\beta ={(\Lambda }_{1}\Lambda_{2}+\Lambda_{5}^{2}-\Lambda_{3}^{2})k_{1}^{2}-\omega^{2}(\Lambda_{2}+\Lambda_{5}),\;\gamma =(\Lambda_{1}k_{1}^{2}-\omega^{2})(\Lambda_{5}k_{1}^{2}-\omega^{2}),\\ \xi_{1}^{2}=-k_{2}^{2}+P_{+},\;\xi_{2}^{2}=-k_{2}^{2}+P_{-},\;\xi_{3}^{2}=-k_{2}^{2}+\left(\omega^{2}-\Lambda_{5}k_{1}^{2}\right)/\Lambda_{4}\\ \Lambda_{1}=C_{11}/\rho ,\;\Lambda_{2}=C_{22}/\rho ,\;\Lambda_{3}=(C_{12}+C_{55})/\rho ,\;\Lambda_{4}=C_{44}/\rho ,\;\Lambda_{5}=C_{55}/\rho ,\;C_{23}=C_{22}-2C_{44} \end{array}$$

$C_{u}^{i}$ and $C_{d}^{i}$ are the displacement amplitude of the wave field signifying the polarity in the media obtained from the eigenvectors for a specific eigen value subjected to specific boundary conditions. Substituting the eigen vectors and then the potential to the Equation (13) wave displacement functions in a transversely isotropic media could be solved. Again, this understanding of the wave modes is crucial to verify and validate numerical or SciML solution results. Before going deeper into SciML, it is customary to recognize the vast research on computational wave filed modeling efforts.

To keep the discussion focused and simplified, following Equations (6), (11) and (12), a generalized wave equation with generalized potential function u is considered. Hence, the wave equation that is discussed is

(18) $$\left(\frac{\partial^{2}u}{\partial {x_{1}}^{2}}+\frac{\partial^{2}u}{\partial {x_{2}}^{2}}+\frac{\partial^{2}u}{\partial {x_{3}}^{2}}\right)=\frac{1}{c^{2}}\frac{\partial^{2}u}{\partial t^{2}}\;\mathrm{or}\;\left(\frac{\partial^{2}u}{\partial x^{2}}+\frac{\partial^{2}u}{\partial y^{2}}+\frac{\partial^{2}u}{\partial z^{2}}\right)=\frac{1}{c^{2}}\frac{\partial^{2}u}{\partial t^{2}}$$

Here, Equation (18) is expressed in terms of *x, y, z*, which corresponds to the Cartesian coordinate system and are equivalent to *x*_1_, *x*_2_, *x*_3_ in the acoustics community. For further discussion, the Cartesian coordinate system is used to represent the spatial coordinates in this study, as PINN studies frequently adopt this notation for computational clarity.

## 3. SciML Architecture: Trends and Transition

With the revolution of machine learning methods, the analysis of large computational or experimental data to catch the patterns, trends, anomalies, or any meaningful scientific insights marks the inception of the concept of SciML [62]. The need for SciML emerged in the late 20th century from the challenges posed by limited labeled or unlabeled, noisy, high-dimensional, and multiscale scientific data. The absence of known ground truth, low human interpretability, and the lack of domain knowledge-based models further exacerbate these challenges [63]. This section explains the root of SciML models and presents how each model is refined over time to fit the physics of engineering problems.

### 3.1. Progression in Physics Infusion

#### 3.1.1. Physics-Guided Neural Network (PgNN)

During the 1980s to 1990s, numerous research efforts explored the future potential of AI in engineering, covering various disciplines like structural engineering [64], mechanical engineering [65], design optimization [66], and others [67,68]. Shukla et al. [69] conducted a comprehensive bibliometric analysis of publications spanning 30 years (1988–2018) on the application of different ML algorithms in engineering. The findings reveal that NN models are the most widely used, underscoring their robustness and effectiveness in these fields [69]. In 1990, Anderson et al. [70] first used an Artificial Neural Network (ANN) to predict direct weld parameters (e.g., bead width, penetration, etc.) from indirect equipment parameters (e.g., welding current, arc voltage) in the Gas Tungsten Arc Welding (GTAW) process. Following this notion, there have been numerous studies where scientists have combined pure physics-driven methods with existing ML models [71,72]. ***The approach of feeding the data from any computational simulation or experimental work to the NN models, where the parent data sets automatically adhere to the physical laws is categorized as Physics-Guided Neural Network (PgNN)* [73]**.

PgNN has contributed to the remarkable advancement of AI [74,75,76,77,78,79,80]. However, this approach has a significant drawback: neural network models provide results without explaining how or why they arrived at them, making it difficult for researchers to identify the cause of errors when they occur. These models are particularly prone to inaccurate predictions when working with small datasets. One possible solution is to increase the amount of training data. However, collecting and processing large datasets can be computationally demanding and often impractical. Additionally, because these models rely solely on data, they struggle to make accurate predictions beyond the range of their training set. This limited ability of generalization reduces their effectiveness when encountering new or unexpected inputs [81]. Daw et al. [82] characterized these models as “physics-agnostic” because although the data are derived from physics simulations or experiments, the model may disregard the fundamental physical laws while training. This is because the NN layers have no information on abiding the physical laws.

#### 3.1.2. Physics-Informed Neural Network (PINN)

To overcome the limitations of the PgNN based approach, researchers [83] introduced Physics-informed Neural Networks (PINNs). This method integrates physical laws with data-driven models to solve engineering problems governed by partial differential equations (PDEs). In PINNs, these physical laws are incorporated into the residuals of neural network models. This innovative approach has gained significant attention across various engineering fields, as reflected in its high citation count within a short period [84]. Since many engineering problems are formulated using PDEs [85], the ability to solve them effectively makes PINNs a valuable tool for engineers. However, this approach has several drawbacks, including susceptibility to overfitting, limited generalization [86], slow convergence, high-dimensional non-convex loss functions [87], and difficulty in simulating large domains [42]. Additionally, PINNs require re-optimization for different boundary and initial conditions, making them inefficient for varying operating conditions and real-time applications [88]. Another major concern is their lack of guaranteed adherence to physical constraints [89].

#### 3.1.3. Neural Operator (NO) and Physics-Encoded Neural Network (PeNN)

In 2019, Lu et al. [90] introduced the Neural Operator (NO) approach, which differs fundamentally from PgNN and PINN by enabling function-to-function mapping. This inherent capability enhances generalization, allowing NOs to predict with input data that are previously unseen, where both PINN and PgNN often struggle. NOs have gained significant popularity within the scientific machine learning (SciML) community, particularly for their ability to handle entire families of PDEs rather than being restricted to solving a single instance [88,91,92,93]. However, this advantage comes at the cost of requiring an extensive amount of training data, leading to high computational overhead. Once trained, however, NOs demonstrate remarkable efficiency, generalizing new cases with minimal or even no additional training [88].

More recently, in 2021, Rao et al. [94] proposed the Physics-encoded Neural Network (PeNN), which takes a different approach by enforcing physical principles directly within the network architecture. Unlike PINNs, where physics is incorporated via the loss function without guaranteeing strict adherence to governing equations, PeNN integrates physical laws more rigorously using advanced neural network formulations. Despite its potential, research on PeNN remains in its early stages, with limited studies exploring its capabilities.

**Figure 3:**
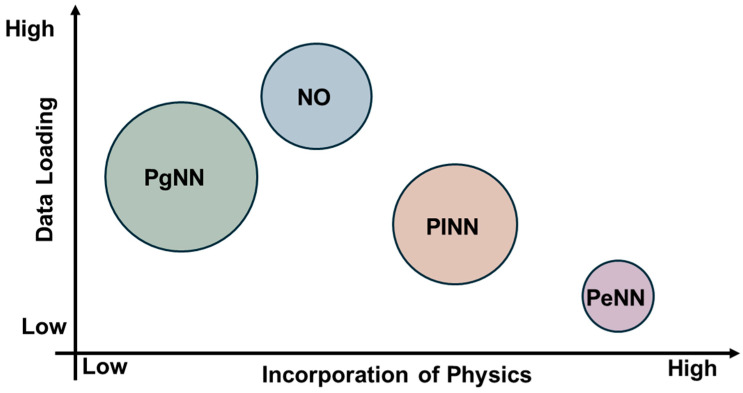
Comparative graph on the dependency on the data and physical laws and existing literature of PgNN, PINN, PeNN, and NO.

Figure 3 illustrates the recent trends across the four modeling approaches discussed. The size of each shape represents the volume of research conducted on each method, highlighting that PgNN, being the earliest, has an extensive body of literature. Among the more recent techniques, PINN remains the most widely adopted. Additionally, a comparative graph is presented to analyze the varying degrees of reliance on data versus physical principles across these models. This discussion naturally leads to a broader classification of modeling approaches into black box, gray box, and white box categories, based on their transparency and dependence on empirical data and governing physics. The next section delves into these classifications, offering deeper insights into their implications for scientific modeling.

### 3.2. Black, Gray, and White Box Concept in SciML

The terms *black box, gray box,* and *white box* are widely recognized across international research communities in machine learning, systems engineering, software testing, and various engineering disciplines [95,96,97,98]. These classifications are based on the level of transparency and interpretability in both the modeling process and the resulting predictions. This section provides an overview of these modeling paradigms, followed by an analysis of the evolution of PgNN, PINN, NO, and PeNN, illustrating the transition from purely data-driven techniques to more advanced models that integrate physical principles and constraints.

*Black Box:* Black box models are characterized by their complex and often opaque decision-making processes, making them difficult to interpret, even for domain experts [98]. Despite this, they exhibit strong predictive performances, making them highly effective for real-world applications. This category includes a diverse range of sophisticated models, such as hyperplane-based techniques like Support Vector Machines (SVMs) [99,100], biologically inspired architectures like Artificial Neural Networks (ANNs) [101], Generative Adversarial Networks (GANs) [102], and Convolutional Neural Networks (CNNs) [103], as well as probabilistic frameworks such as Markov Networks [104] and Bayesian Networks [105,106]. Additionally, instance-based learning methods like k-Nearest Neighbors (k-NN) [107] also fall under this classification.

*White Box:* In contrast to the black box modeling approach, white box models, also known as explainable artificial intelligence (XAI) models, prioritize transparency, interpretability, and explainability in the decision-making processes [108]. Among the pioneering white box models, decision tree-based models stand out for their straightforward and easily interpretable structure [109]. Additionally, rule-based [110] and pattern-based models [111] also fall under the white box category. Though the white box models are great in the case of transparency, they mostly show lower accuracy than the black box models [98]. These models are predominantly used for classification problems in the field of medicine and finance. Conversely, black box models are widely used to address engineering problems [98].

**Figure 4:**
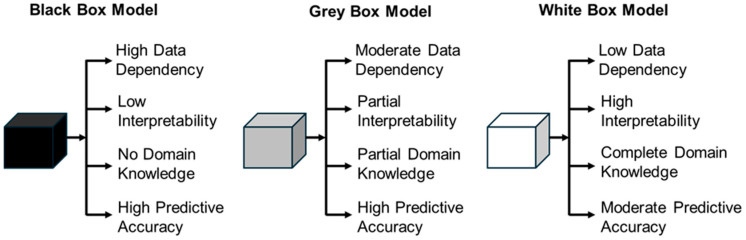
Comparative analysis of black box, gray box, and white box modeling.

*Gray Box:* The gray box modeling represents a hybrid approach that combines the black box models and white box models to utilize the advantages of both methods in an optimized manner. This approach allows for a more nuanced understanding of complex systems, making it particularly valuable in engineering fields where both accuracy and explainability are crucial. This particular synergy bridges the gap between purely data-driven approaches and theoretical knowledge [112]. Some established methods under this category include PINN [83], adaptive network-based fuzzy inference system [113], PeNN [94], etc. Figure 4 represents a comparative analysis of the black, gray, and white box modeling approach.

**Figure 5:**
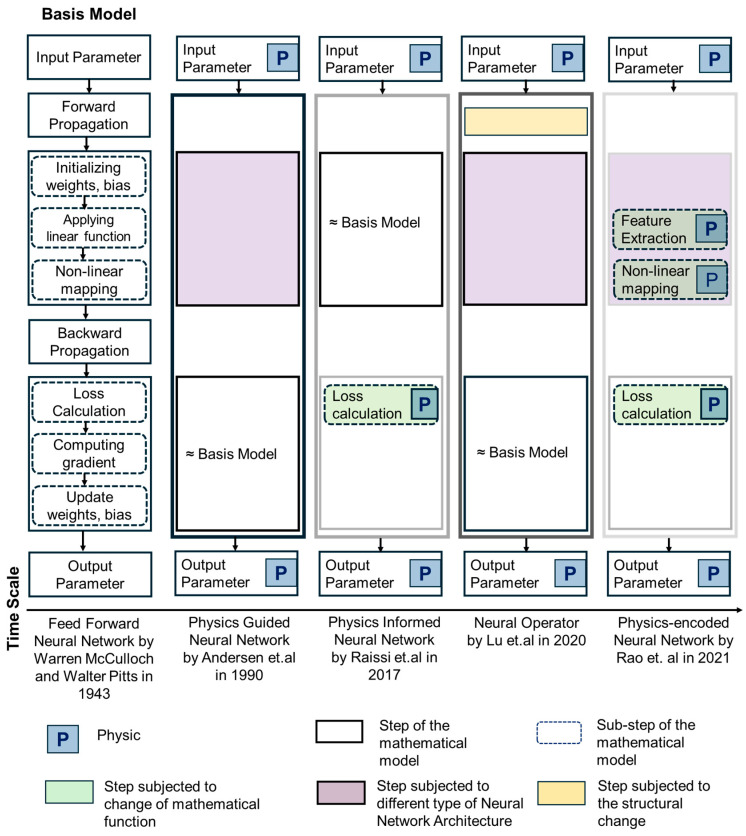
A schematic representation illustrating the evolution of the traditional ANN architecture by McCulloch and Pitts over time, focusing on the integration of physics [117]. The first approach to incorporate physics is the PgNN model, where the input and output data are constrained by physical laws [70]. Various advanced NN architectures have been explored within this framework. The subsequent SciML method, PINN, was introduced by Raissi et al. [83]. In PINN, forward propagation follows the same mechanism as in traditional ANN, but the loss function is modified to embed physical laws. Later, NOs were proposed by Lu et al. [90], introducing structural changes to forward propagation. These changes include simultaneous forward propagations or the incorporation of advanced techniques such as Fourier transform, Wavelet transform, or Green’s function. Despite these modifications, backpropagation in NOs remains similar to that of ANN. The most recent development is the PeNN model [94], which offers flexibility in adopting different NN architectures and advanced approaches for embedding physical laws in forward propagation. The method for calculating the residual function in PeNN aligns with the approach used in PINN. Finally, the color-coded boxes for each SciML method represent their classification as black, gray, or white Box models, emphasizing their level of transparency and reliance on data and physics.

Building upon the historical progression outlined in Section 3.1, it is evident that the evolution of Scientific Machine Learning (SciML) has been driven by the continuous effort to enhance AI-based modeling techniques for complex engineering challenges. Figure 5 captures this trajectory, illustrating how fundamental modifications to neural network architectures have enabled the seamless integration of physical principles.

The initial phase of SciML primarily relied on incorporating physics-derived data into machine learning models. Over time, this approach evolved to fuse both experimental data and governing equations, reinforced by physical constraints. More recent advancements have moved beyond this hybridization, embedding mathematical constructs such as Fourier transforms [114], Green’s functions [115], and Wavelet transforms [116] directly into neural network frameworks. These enhancements mark a critical transition from purely data-driven or equation-based approaches toward a more balanced, physics-informed paradigm, ensuring greater accuracy and adaptability in engineering applications.

### 3.3. SciML Frameworks for Wave Propagation

This section begins with an introduction to fundamental artificial neural networks, establishing a foundational understanding before gradually transitioning to more advanced models. Each model’s algorithm is thoroughly examined in the context of wave propagation, as governed by Equation (18).

#### 3.3.1. Artificial Neural Network (ANN)

ANNs are computational models inspired by the concept of the human brain. In 1943, McCullough and Pitts proposed the theoretical idea of neural computation [117]. By 1958, Rosenblatt proposed the concept of the Feed Forward Neural Network (FFNN) [118]. FFNN is the simplest form of ANN where the information moves in one direction. An FFNN consists of an input layer, one or more hidden layers, and an output layer. Each layer contains neurons, and every neuron is connected to the neurons in the next layer. Neurons are the basic computational units of the network. Equation (19) represents the mathematical formulation of the FFNN.

(19) $$Y=\sigma \left(wX+b\right)$$

**Figure 6:**
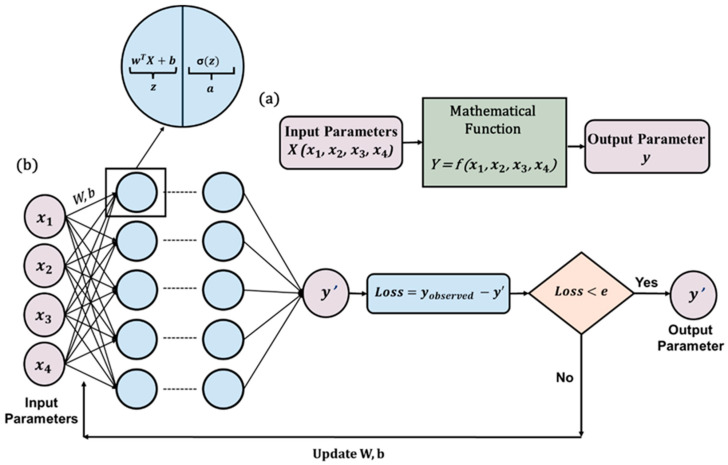
A schematic architecture of ANN. (**a**) Generation of training data using a known mathematical function. (**b**) The architecture of an ANN model consisting of a simple feed-forward network. Here, y’ represents the predicted output. Until the loss (loss can be calculated using MSE, RMSE, etc.) becomes less than the predefined tolerance value e, the model keeps updating the weight (*w*) and bias (*b*) parameters iteratively. Idea depicted from [59].

In this equation, X and Y subsequently denote the input and output of the neuron, w represents the weight parameter, and b represents the bias parameter as well as the activation function. The activation function introduces non-linearity to the forward propagation system. This approach was not fruitful enough until backpropagation was introduced in 1980 [119]. The backpropagation algorithm is used to train the FFNN. Training of the FFNN starts with calculating the discrepancy between the predicted and actual value of the output. This is called loss function (L). In the backpropagation, the gradients $\frac{\partial L}{\partial w}$ and $\frac{\partial L}{\partial b}$ of the loss function, with respect to each of the weight and bias parameters of the network, are calculated. The weights and bias parameters are updated using optimization algorithms based on the gradients. Figure 6 depicts the architecture of the ANN.

#### 3.3.2. Physics-Guided Neural Network (PgNN)

The PgNN framework is widely utilized for modeling wave equations, demonstrating its applicability in capturing wave propagation dynamics. Adam R. Brink et al. [120] employed a feedforward multilayer perceptron to predict displacement fields based on spatial and temporal coordinates. While this approach provided valuable insights, the limitations of a simple FFNN led researchers to explore more advanced neural network architectures to better address the complexities of wave equations [121,122,123,124,125,126].

**Figure 7:**
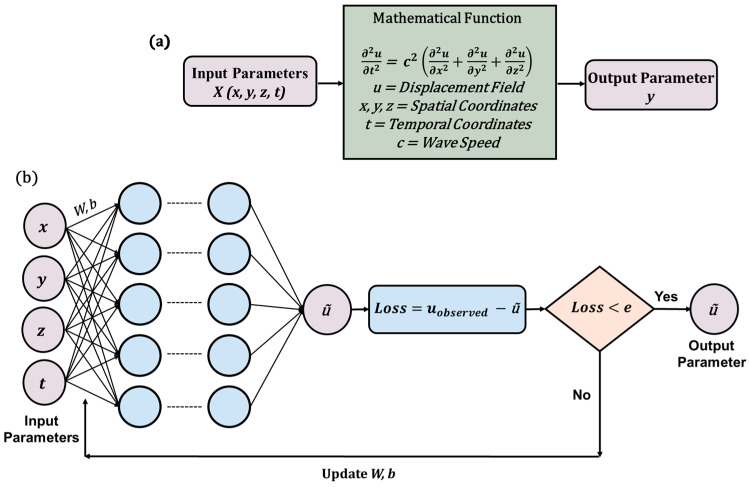
An illustration of the PgNN model. (**a**) Generation of training data by solving the wave equation using the traditional approach. (**b**) The architecture of the PgNN model consists of a simple feed-forward network. Here, the spatial–temporal coordinates (x,y,z,t) are the input parameters into the model. Based on the given input parameters, the model predicts the displacement field $\tilde{u}$. Until the loss (loss can be calculated using MSE, RMSE etc.) becomes less than the predefined tolerance value e, the model keeps updating the weight (*w*) and bias (*b*) parameters iteratively. Idea depicted from [59].

For clarity, this section presents PgNN using the FFNN framework. Figure 7 provides a schematic representation of PgNN, a data-driven model that relies entirely on input data. Here, the independent variables (*x*, *y*, *z*, *t*) serve as input parameters, and the model predicts the displacement field as the output. The underlying mathematical formulation follows a similar approach to that discussed in Section 3.3. The training of this deep learning model can be performed using either simulated or experimental datasets, adhering to a supervised learning paradigm.

#### 3.3.3. Physics-Informed Neural Network (PINN)

Among the various SciML approaches, PINN has emerged as the most widely adopted model. The foundational framework was first introduced [84] through the development of the vanilla PINN. The core principle of PINN lies in reformulating the regularization term to embed physical laws directly into the learning process. This section provides a detailed explanation of the PINN algorithm, specifically in the context of solving wave equations. The key distinction between PINN and the previously discussed PgNN model lies in the formulation of the loss function.

In PgNN, the loss function is defined solely by the discrepancy between the observed and predicted displacement fields, relying only on displacement data at known spatial–temporal coordinates without additional constraints. In contrast, PINN incorporates governing differential equations along with initial and boundary conditions (I/BCs) to refine its predictions. This results in three additional loss terms that enforce physical consistency. To illustrate the formulation of the loss function, this paper considers a 3D wave equation, as expressed in Equation (18), subject to Dirichlet boundary conditions and initial conditions. The solution domain is represented as a simple 3D box, defined by $x_{b}\in \left[a_{x},b_{x}\right],\;y_{b}\in \left[a_{y},b_{y}\right]$, and $z_{b}\in$ $\left[a_{z},b_{z}\right]$. The boundary points of this domain are denoted as Ω, with the corresponding Dirichlet boundary conditions given in Equation (20).

(20) $$\left.\begin{array}{ll} u\left(a_{x},y,z,t\right)=0, & u\left(b_{x},y,z,t\right)=0\\ u\left(x,a_{y},z,t\right)=0, & u\left(x,b_{y},z,t\right)=0\\ u\left(x,y,a_{z},t\right)=0, & u\left(x,y,b_{z},t\right)=0 \end{array}\right\}$$

These conditions indicate that the displacement at the boundaries in all possible directions is always zero. The initial conditions for displacement and velocity terms over the entire domain are expressed in Equation (21). Here, f(x,y,z) and g(x,y,z) are the initial displacement and velocity field at t=0. The set of data points for initial conditions are expressed by Ψ.

(21) $$\left.\begin{array}{c} u(x,y,z,0)=f(x,y,z)\\ \frac{\partial u}{\partial t}(x,y,z,0)=g(x,y,z) \end{array}\right\}$$

With the solution domain established and the initial and boundary conditions (I/BCs) specified, the loss functions for the PINN model are expressed in Equations (22)–(26).

(22) $$L=\lambda_{a\;}L_{data}+\lambda_{b\;}L_{PDE}+\lambda_{c\;}L_{BC}+\lambda_{d\;}L_{IC}$$

(23) $$L_{\mathrm{data}}=\frac{1}{N}\sum_{i=1}^{N}\;{\left|\tilde{u}\;\left(x_{i},y_{i},z_{i},t_{i}\right)-u_{i}^{obs}\right|}^{2}$$

(24) $$L_{PDE}=\frac{1}{M}\sum_{j=1}^{M}\;{\left|\frac{\partial^{2}\tilde{u}\;\left(x_{j},y_{j},z_{j},t_{j}\right)}{\partial t^{2}}\;\;\;-c^{2}\left(\frac{\partial^{2}\tilde{u}\;\left(x_{j},y_{j},z_{j},t_{j}\right)}{\partial x^{2}}+\frac{\partial^{2}\tilde{u}\;\left(x_{j},y_{j},z_{j},t_{j}\right)}{\partial y^{2}}+\frac{\partial^{2}\tilde{u}\;\left(x_{j},y_{j},z_{j},t_{j}\right)}{\partial z^{2}}\right)\right|}^{2}$$

(25) $$L_{BC}=\frac{1}{K}\sum_{k=1}^{K}\;{\left|\tilde{u}\;\left(x_{k},y_{k},z_{k},t_{k}\right)\right|}^{2}$$

(26) $$L_{IC}=\frac{1}{M}\sum_{j=1}^{M}\;\left({\left|\tilde{u}\;\left(x_{j},y_{j},z_{j},0\right)-f\left(x_{j},y_{j},z_{j}\right)\right|}^{2}+{\left|\frac{\partial \tilde{u}\;\left(x_{j},y_{j},z_{j},0\right)}{\partial t}-g\left(x_{j},y_{j},z_{j}\right)\right|}^{2}\right)$$

**Figure 8:**
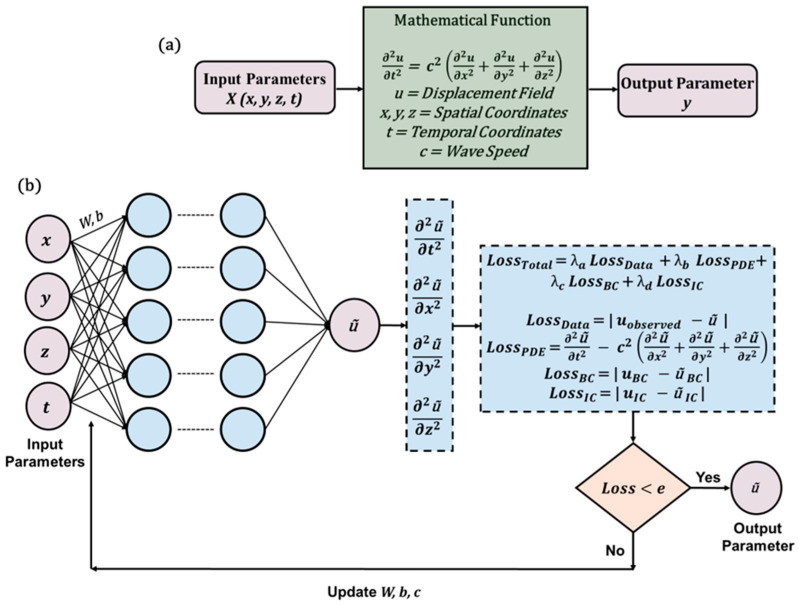
An illustration of PINN model architecture. (**a**) Generation of training data by solving the wave equation using traditional approach. (**b**) The architecture of the PINN model. Here, $\tilde{u}$ represents the predicted displacement field. By using automatic differentiation, the gradients of the predicted displacement field have been calculated which is later used for calculating the Loss_PDE._ The loss function has four different components: ${Loss}_{\mathrm{Data}\;}$, ${Loss}_{\mathrm{PDE}}$, ${Loss}_{\mathrm{BC}\;}$, and ${Loss}_{\mathrm{IC}\;}$. Until the loss becomes less than the predefined tolerance value *e*, the model keeps updating the weight (w) and bias (b) parameters along with the unknown parameter c iteratively. Idea depicted from [59].

Here, the loss term $L_{\mathrm{data}}$ follows the same structure as in the PgNN model. ${\left\{\left(x_{i},y_{i},z_{i},t_{i},u_{i}^{\mathrm{obs}}\right)\right\}}_{i=1}^{N}$ represents the spatial–temporal coordinates, where $u_{i}^{\mathrm{obs}}$ denotes the observed values at the i−th point $\left(x_{i},y_{i},z_{i},t_{i}\right)$ inside the domain. The additional three terms $L_{\mathrm{PDE}}$, $L_{\mathrm{BC}},\;{andL}_{\mathrm{IC}}$ incorporate the underlying physics. The $L_{\mathrm{PDE}}$ term enforces the governing partial differential equation (PDE) at a set of collocation points ${\left\{\left(x_{j},y_{j},z_{j},t_{j}\right)\right\}}_{j=1}^{M}$ within the domain. The indices i for an observed point and the indices j for a collocation point may or may not correspond. Similarly, $L_{\mathrm{BC}}$ and $L_{\mathrm{IC}}$ enforce the boundary and initial conditions, respectively. The set ${\left\{\left(x_{k},y_{k},z_{k},t_{k}\right)\right\}}_{k=1}^{K}$ defines additional collocation points where these constraints apply. The weightage factors $\lambda_{a\;}$, $\lambda_{b\;}$, $\lambda_{c\;}$, and $\lambda_{d\;}$ determine the relative importance of each loss term. Figure 8 provides a schematic representation of the PINN architecture, and Algorithm 1 presents the pseudocode for the vanilla PINN applied to solving the wave equation.

This approach is known as the soft enforcement of physics that integrates physical laws through the regularization term. Embedding physics into the learning process expands possibilities for tackling problems where labeled data is scarce or entirely unavailable. Researchers continue to investigate whether this method can produce reliable results without labeled data, and so far, the findings are promising. This framework also demonstrates robustness when handling sparse or noisy data.

One of the most compelling advantages of this approach is its capability to address inverse problems effectively. This potential has driven the researchers to explore both forward and inverse problems within the same framework. The forward problem involves predicting system behavior using known parameters and embedded physics, while the inverse problem focuses on determining unknown parameters from observed data. PINNs unify these two approaches under a single optimization process, enabling simultaneous solutions for both.

**Figure 9:**
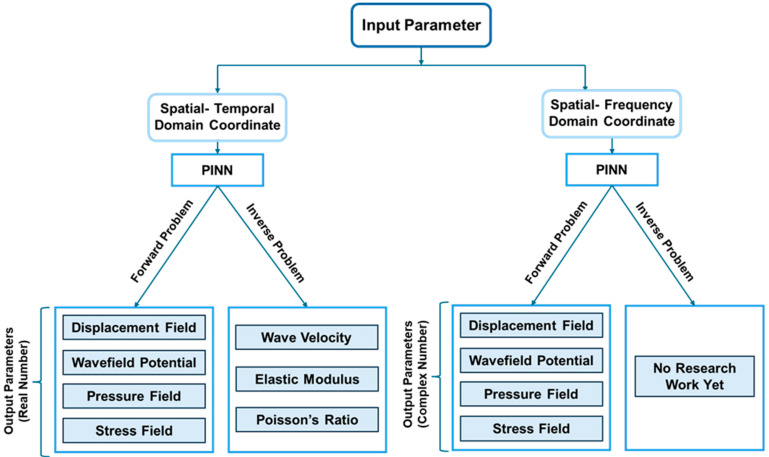
A schematic representation of forward and inverse problems using relevant parameters in PINN architecture for wave propagation.

In recent years, researchers have successfully applied PINNs to solve wave equations in both time and frequency domains [127,128,129]. In frequency domain problems, instead of using time as an input variable, frequencies serve as the primary input parameters. A key challenge in this domain arises from the fact that neural networks are not inherently designed to handle complex numbers. To circumvent this limitation, the model processes the real and imaginary components separately. Figure 9 illustrates the distinction between time domain and frequency domain inputs, along with the corresponding outputs for forward and inverse problems in the context of wave propagation modeling.
**Algorithm 1:** PINNRequire:   1: Ω: Set of boundary points of the domain   2: Ψ: Set of initial points of the domain   3: u: Actual output from the PDE equation   4: c: Constant   $\;5:\;\lambda_{a\;},\;\lambda_{b},\lambda_{c\;},\lambda_{d\;}$: Loss weights   6: T: Maximum training iterationsEnsure:   7: Trained neural network NN   8: procedure Train_PINN   9:      for τ = 1 to T do   10:         $\tilde{u}$ = NN ($x_{i},y_{i},z_{i},t_{i}$)   11:         $L_{data}$  ←   $\frac{1}{N}\sum_{i=1}^{N}\;{\left|\tilde{u}\left(x_{i},y_{i},z_{i},t_{i}\right)-u_{i}^{obs}\right|}^{2}$   12:         $residual_{PDE}$  ←   $\frac{\partial^{2}\tilde{u}}{\partial t^{2}}-c^{2}\left(\frac{\partial^{2}\tilde{u}}{\partial x^{2}}+\frac{\partial^{2}\tilde{u}}{\partial y^{2}}+\frac{\partial^{2}\tilde{u}}{\partial z^{2}}\right)$   13:         $L_{PDE}\leftarrow \frac{1}{M}\sum_{j=1}^{N}\;$($residual_{PDE}$)^2^   14:         $residual_{BC}$ ←  ${\tilde{u}}_{bc}$ − NN ($x_{i},y_{i},z_{i},t_{i}$)    where {($x_{i},y_{i},z_{i},t_{i}$)} ϵ Ω   15:         $L_{BC}\;\leftarrow \frac{1}{K}\sum_{k=1}^{K}\;{\left(residual_{BC}\right)}^{2}$   16:         $residual_{IC}\leftarrow {\tilde{u}}_{ic}-NN\left(x_{i},y_{i},z_{i},t_{i}\right)\;where\{(x_{i},y_{i},z_{i},t_{i})\}\epsilon \Psi$   17:         $L_{IC}\;\leftarrow \frac{1}{M}\sum_{M=1}^{M}\;{\left(residual_{IC}\right)}^{2}$   18:         $L\;\leftarrow =\lambda_{a\;}L_{data}+\lambda_{b\;}L_{PDE}+\lambda_{c\;}L_{BC}+\lambda_{d\;}L_{IC}$   19:         Update NN using L trough backpropagation.   20:         **if** L ≈ 0 **then**   21:              **Break**   22:         **end if**   23:      **end for**   24:      Output trained neural network NN   25: **end procedure**


#### 3.3.4. Neural Operator (NO)

Most existing approaches provide solutions for a specific PDE with predefined initial and boundary conditions (I/BCs). Once trained, these models cannot generate solutions for the same PDE under different I/BCs without retraining. For instance, PINN, PgNN, and PeNN solve only one particular set of I/BCs for a given PDE, requiring a separate training process for each new scenario [88]. Neural Operators (NOs) offer an innovative solution to bypass this limitation. Lu et al. [90] introduced the first NO model, DeepONet, in 2019. Since then, several variations have emerged, including the Fourier Neural Operator (FNO) [114] and the Wavelet Neural Operator (WNO) [116]. These advanced models are discussed in detail in Part 2 of this article, while this section focuses on the architecture of DeepONet within the context of wave propagation.

**Figure 10:**
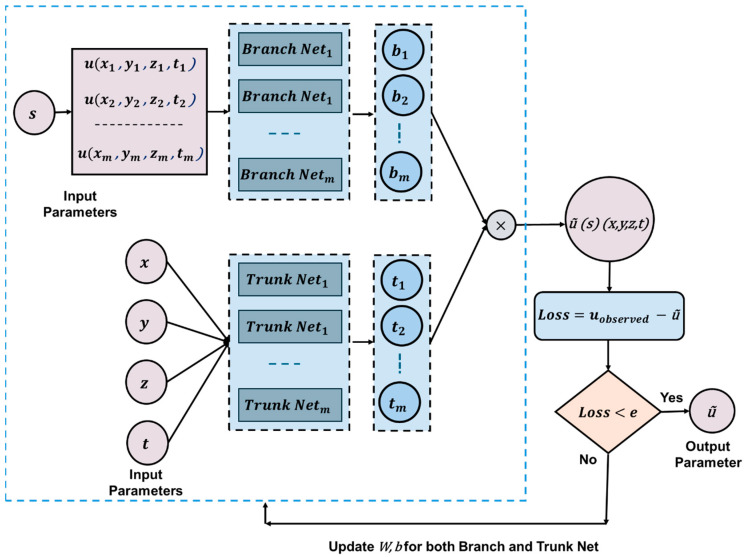
An architecture of NO (DeepONet). The DeepONet model has two components: branch network and trunk network. These networks may consist of one or more NN models in stacked or unstacked manners. The branch network takes *s* as the input parameter. Here, *s* denotes different functions representing various I/BCs. The trunk network takes the spatial–temporal coordinates (*x*, *y*, *z*, *t*). These two networks extract the feature information from the input data. The output of these two networks later multiplied to get the final output which is the displacement filed $\tilde{u}$. Till the loss (loss can be calculated using MSE, RMSE, etc.) becomes less than the predefined tolerance value *e*, the model keeps updating the weight (*w*) and bias (*b*) parameters iteratively for both branch and trunk network [114].

Figure 10 illustrates the DeepONet architecture, which consists of two primary networks: the branch network and the trunk network. The branch network processes the input function, extracting critical features from the data to understand the behavior of the solution function across different I/BCs. Any deep neural network model can serve as the feature extractor within this branch network. Meanwhile, the trunk network takes spatial–temporal coordinates as input, defining the specific locations where the solution is evaluated—referred to as sensors. The final output is obtained through a dot product operation between the outputs of the branch and trunk networks. The model then compares this prediction with observed data to compute the loss function. Similar to standard artificial neural networks (ANNs), DeepONet updates its weights and biases through backpropagation during the training process.

#### 3.3.5. Physics-Encoded Neural Network (PeNN)

Rao et al. [94] introduced the physics-encoded learning process in 2021, enforcing physical laws through a coercive mechanism. While the fundamental idea is not entirely new, their approach to embedding physics marks a paradigm shift. The model incorporates advanced neural network architectures such as Convolutional Neural Networks (CNNs) and Recurrent Neural Networks (RNNs). Instead of traditional activation functions, the authors introduced elementwise product operations to introduce non-linearity. The method was tested on the 2D Burgers equation and the 3D Gray–Scott (GS) reaction–diffusion (RD) equation. However, no studies have yet applied this approach to solving the wave equation. This section explores the architecture of the Physics-encoded Neural Network (PeNN) within the context of wave modeling.

**Figure 11:**
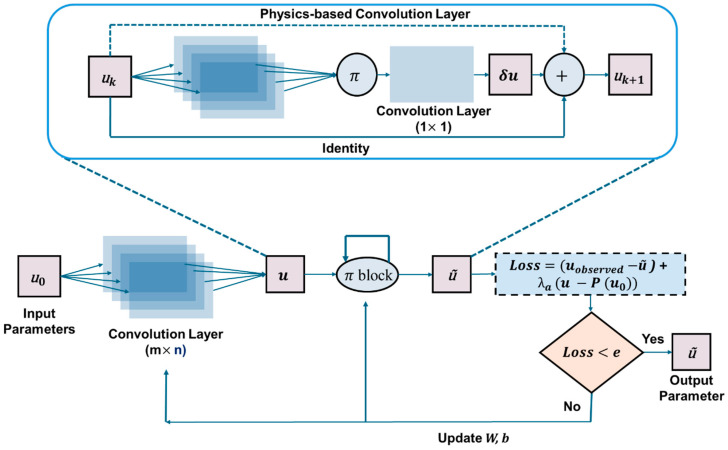
An architecture of PeNN (PeRCNN). The network works in two parts: Initial State Generator (ISG) and π-block. The network here first takes the low-resolution noisy initial state displacement field u_0_ as the input parameter_._ The data is then passed through traditional convolutional layers, which results in full resolution displacement field *u*. This data is then fed into the π-block. Here, $u_{k}$ represents the full resolution displacement field at the time step k. Then, this data goes through multiple parallel convolutional layers again. The output of each convolutional layer is then combined by elementwise multiplication. Later, the product is passed through a (1×1) convolutional layer, which results in $\delta {\hat{u}}_{k}$. The final outcome is the displacement field $u_{k+1}$ at the (k+1) time step, which is the resultant of the elementwise addition of $u_{k}$ and $\delta {\hat{u}}_{k}$. The loss function is calculated for both the initial state generator and the π-block part. In the loss function, p represents the spatial interpolation function. Until the loss becomes less than the predefined tolerance value *e*, the model keeps updating the weight (*w)* and bias (*b*) parameters iteratively for all the convolutional layers in ISG and π-block [94].

The proposed framework consists of two main components. The first, termed the Initial State Generator (ISG), generates an initial-resolution state from a noisy, low-resolution measurement of the state variable $u_{0}$. This process utilizes a fully convolutional layer for feature extraction. The second component, known as the π-block, carries the state variable $u_{k}$ from the previous time step. This block employs multiple convolutional filters to extract relevant features from the data. Instead of applying a traditional activation function, the method introduces non-linearity through elementwise multiplication. The resulting output is then passed through a (1×1) convolutional filter, producing $\delta {\hat{u}}_{k}$. The final state variable, $u_{k+1}$, is obtained by elementwise addition of $\delta u_{k}$ and $u_{k}$. In this framework, boundary conditions are satisfied through a padding mechanism, while initial conditions are enforced via the first input state variables. Figure 11 illustrates a schematic representation of the PeRCNN architecture.

## 4. Applications of PINN in Wave Propagation

**Figure 12:**
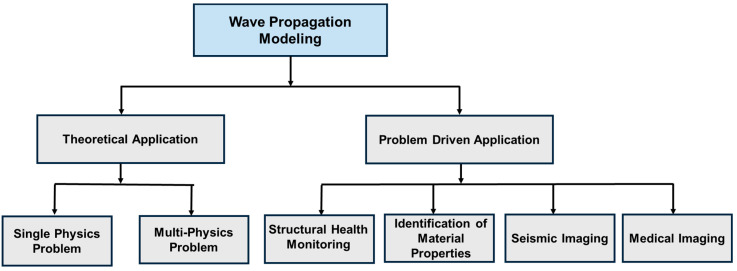
Classification tree on the application-based work of wave propagation modeling with PINN.

PINNs offer significant advantages in wave propagation modeling compared to conventional machine learning models. By integrating physics-based constraints, PINNs reduce dependence on large labeled datasets and ensure physically consistent solutions [130]. This section categorizes wave propagation problems into theoretical and problem-driven applications, providing a structured overview of PINN’s diverse implementations. The classification tree in Figure 12 illustrates this categorization.

Theoretical applications focus on refining traditional PINN frameworks to handle the complexities of solving wave equations. Additionally, research in this area explores the integration of wave equations with other PDEs to model multi-physics phenomena. Section 4.1 and Section 4.2 detail studies on solving standalone and coupled wave equations, respectively. Table 1 presents a curated selection of recent studies that utilize PINNs for wave equation solutions, showcasing innovative techniques addressing various computational challenges.

In problem-driven applications, researchers have applied PINNs to model acoustic and elastic wave propagation across four key research areas: (a) structural health monitoring, (b) material property identification, (c) seismic imaging, and (d) medical imaging. Section 4.3–Section 4.6 provide an in-depth review of research efforts in these fields. Table 2 summarizes recent studies leveraging PINNs for problem-solving in these applications.

### 4.1. Single Physics Problem

PINNs, being a mesh-free algorithm, have gained significant importance among researchers in the field of wave propagation. Since its introduction, the application of PINNs in wave propagation problems has been an active area of research. In 2020, Guo et al. [15] first used PINN to model the 1D wave equation for a small spatial and temporal range. The study considered homogeneous Dirichlet boundary conditions and initial conditions, using approximately 4000 labeled data points for training the model. Despite employing a vanilla PINN, the results showed strong agreement with the ground truth. However, a key limitation of this approach was the extensive requirement for labeled data, which contradicts one of the fundamental motivations for adopting PINNs—reducing dependence on labeled datasets. Building on this foundation, Alkhadhr et al. [131] expanded the problem scope in 2021 by introducing a source term into the wave equation and extending the domain of simulation to $(x\in \left[0,\;5\right],\;t\;\in \left[0,5\right]$). Validation was performed using numerical solutions obtained via the Finite Difference Method (FDM). The study demonstrated that, for this particular problem, PINNs achieved a 47% reduction in computational time compared to conventional FDM techniques. Both studies utilized the hyperbolic tangent function as the activation function and employed the ADAM and Limited-memory Broyden–Fletcher–Goldfarb–Shanno-Bound (L-BFGS-B) optimizers for training.

In 2022, Dania Sana [132] conducted an in-depth study on the optimization strategies for PINN-based wave equation modeling. Through rigorous error analysis, the study demonstrated that training with only the ADAM optimizer resulted in slow convergence, whereas incorporating L-BFGS-B improved both convergence speed and stability. Several critical aspects of solving wave equations using PINNs were introduced, including training the model with approximately 200 samples for the 1D wave equation under Dirichlet boundary conditions. The work further explored Neumann boundary conditions, wave equation degeneration scenarios where wave velocity was treated as a function of spatial variables or constants, and saw the first application of PINNs to inverse problems and null boundary control problems.

Although the proposed PINN framework largely followed the vanilla PINN structure, it incorporated an adaptive loss weighting scheme determined through a trial-and-error approach. Compared to previous studies, this work required fewer training samples but remained constrained to a relatively small simulation domain.

That same year, Wu et al. [133] introduced significant modifications to improve the modeling of the wave equation using PINNs. Several key challenges associated with solving wave equations were addressed, including source singularity—where discontinuities or sharp spikes in the source term cause numerical instability and inaccuracies [134]. Another major drawback of the vanilla PINN is its slow convergence rate and reduced accuracy when handling sharp contrasts in the PDE of the scattered wavefield.

To mitigate the issue of source singularity, the research group opted to solve the wave equation in the frequency domain rather than the time domain. The study also incorporated a Perfectly Matched Layer (PML) into the loss function, an artificial absorbing boundary condition designed to eliminate reflections from the domain edges [133]. By incorporating PML, the neural network effectively predicted the real part of the wavefield solution from the source to the boundary, while also reconstructing the imaginary part with high accuracy.

Additionally, instead of conventional neurons, the authors utilized quadratic neurons, which introduced greater non-linearity into the model. This enhancement allowed the network to better capture the non-smooth, complex Laplacian of the wavefield, leading to improved performance and stability [135].

Other studies have also focused on overcoming the slow convergence of the PINN model in realistic wavefield simulations. Nosrati and Niri [87] identified the multi-term objective function as a key factor contributing to the slow convergence issue. They conducted a failure analysis of the PINN model and visualized the loss landscape. In addition to slow convergence, the oscillatory nature of the wave equation’s solution presents another major challenge for researchers. This behavior becomes evident when the angular frequency remains constant but the wave velocity decreases, signaling wave dispersion. To address these issues, the authors proposed using a logarithmic loss function (PDE). Their analysis revealed that the model converges more quickly when the weight factor (λ) of the logarithmic loss (PDE) is kept below 1. They also introduced a sigmoid function of λ as a multiplier for the logarithmic loss (PDE), effectively addressing the convergence and oscillatory behavior with an initial increase followed by a gradual decrease in the loss (PDE).

Around the same time, Moseley et al. [42] introduced a new variant called fb-PINN (Finite Basis-PINN), inspired by the traditional Finite Element Method. This model was shown to be more effective for wave propagation in larger domains, an area where the traditional PINN model struggles due to increasing optimization complexity and spectral bias. While much of the prior work focused on small domains, Moseley et al. [42] addressed the optimization challenge by breaking the domain into overlapping subdomains, each handled by a separate neural network. They recommended using parallel computation for faster training and proposed separate normalization of input variables for each subdomain to mitigate spectral bias. Additionally, they employed ansatz functions as hard constraints to ensure the model strictly adheres to the physical laws.

In another study, Alkhadhr and Almekkawy [136] explored the concept of hard and soft enforcement in the context of PINNs. Soft constraints refer to the approach used in the vanilla PINN, where physical laws and constraints are integrated into the regularization terms. Many researchers have raised concerns about this ill-posed method, as it does not guarantee full satisfaction of boundary conditions (BCs) and initial conditions (ICs), since the loss term does not necessarily result in zero. In response, the authors proposed a hard enforcement approach for BCs and ICs within the neural network architecture, which ensures stricter adherence to physical laws. This approach, often referred to as physics-constrained, is considered a variation of the PINN [137].

In solving the 1D wave equation, four combinations of constraints were tested: (1) both ICs and BCs as hard constraints (hard–hard), (2) both ICs and BCs as soft constraints (soft–soft), (3) ICs as hard constraints and BCs as soft constraints (hard–soft), and (4) ICs as soft constraints and BCs as hard constraints (soft–hard). The results indicated that for this particular wave equation case study, the best outcome was achieved with the soft–soft constraints.

Additionally, Chen et al. [138] introduced a variant called HWPINN (Hard Constraint Wide Body PINN) for solving the 2D wave equation. The study compared four models: HPINN (Hard Constraint PINN), HWPINN, SPINN (Soft Constraint PINN), and SWPINN (Soft Constraint Wide PINN), with the term “wide” indicating that more neurons are used per hidden layer. According to their findings, HWPINN outperformed the other models in solving the 2D wave equation.

### 4.2. Multiphysics with Wave Equation

Multiphysics problems are often addressed through coupled PDEs, but solving these equations presents numerous challenges due to the interactions between physical variables, their interdependencies, complex geometries, and higher dimensionality [139,140]. Several studies have explored the use of PINNs for solving coupled PDEs, with thermoelasticity being one key multiphysics phenomenon. Thermoelasticity involves the coupling between elastic wave physics and heat equations, but research applying PINNs to this area remains limited.

Eshkofti and Hosseini [139] investigated 2D thermoelastic wave propagation in a thick hollow cylinder using PINNs. They based their formulation on the Green–Naghdi theory of coupled thermoelasticity, which effectively captures the true behavior of thermoelastic wave propagation in solids. In this study, the displacement field and temperature distribution were modeled using separate neural network architectures. The authors applied the hard constraint method to incorporate Dirichlet boundary conditions and trained the model without any labeled data, relying solely on the coupled PDEs and I/BCs.

In a different study, non-Fickian diffusion in thermoelastic analysis was examined using gradient-enhanced PINNs (gPINNs). The key difference between gPINNs and traditional PINNs is the inclusion of an additional term in the loss function, named Loss__grad_PDE_, which ensures the gradient of the loss function satisfies the independent variables. The results showed a global relative error between 1.9% and 2.8%, although at the cost of significantly increased computational time—doubling or tripling the time required. The authors trained three distinct NN architectures for three different output variables, eventually combining hard and soft encoding of physics in the final model through trial and error [141].

Fang et al. [142] used PINNs to solve the thermoelastic coupling problem for a spherical ice particle. They applied different weightage factors to the vanilla PINN without altering its basic structure. The model achieved an impressive maximum accuracy of 99.99%, with L2 relative errors approaching zero in the temperature field, while errors in the displacement field were slightly larger. Additionally, the model demonstrated a 76.41% reduction in computation time compared to the Finite Element Method (FEM).

**Table 1:** A selective list of studies that includes modifications of PINN model addressing challenges modeling wave equation.

Author	K Issues Addressed	Proposed Modifications
Sana [132]	Error analysis based on the usage of different optimizers Strong and weak degeneration of wave equation Null boundary control problem	Adding weightage factor to the loss term by trial-and-error analysis
Wu et al. [135]	Large domain problem Source singularity Non-smooth velocity model	Solving frequency domain scattered wavefield Quadratic neuron Perfectly matched layer
Nosrati and Emami Niri [87]	Multi-term objective function Oscillatory nature of solution	Self-adaptive logarithmic loss term
Moseley et al. [42]	Large domain Spectral bias	Finite Basis PINN (fb-PINN): Overlapping subdomain decomposition Separate input variable normalization
Alkadhr and Almekkawy [136]	Lack of surety of the adherence of the physical laws	Combination of hard and soft constraints
Chen et al. [138]	Higher dimensional equation	Hard Constraint Wide Body PINN (HWPINN): Hard enforcement of physics, more neurons per hidden layer
Eshkofti and Hosseini [141]	Coupled PDE Curse of dimensionality (COD)	Gradient Enhanced PINN (gPINN): Additional term in the loss function including gradient of the PDE loss

### 4.3. NDE and Structural Health Monitoring

The importance of monitoring structural performance, assessing damage, and estimating the remaining service life of aging structures has been recognized since the early stages of engineering. Over the past two decades, structural health monitoring (SHM) in real time has become a critical area within nondestructive testing and evaluation (NDT&E) [11]. Two approaches emerged as the focus of research in developing an effective SHM system. The global approach centers on analyzing damage-induced vibrational properties, such as modal frequencies and mode shapes, of the system. Global approach of SHM is a passive method. In contrast, the local approach monitors and analyzes actively excited waves and its propagation to detect existing or newly formed defects by observing changes in wave properties [143]. This section reviews studies in which researchers applied the PINN model to analyze wave propagation for diagnosing material damage.

In 2020, Shukla et al. [144] were the first to use PINNs to analyze a surface-breaking crack in a metal plate, with a focus on solving the inverse problem. The study used wave speed as a key indicator for detecting the crack. The model was trained with experimental data collected using the laser vibrometer with three-point laser measurement giving three displacements in three orthogonal directions. Before feeding the data into the model, it was preprocessed using principal component analysis (PCA). To enhance optimization and speed up convergence, the study employed an adaptive activation function. Additionally, the team conducted an analysis to determine the optimal number of training data points needed for the best results.

In a separate study, Ghellero [145] proposed a PINN model combined with a finite difference solver to localize damage in a string (1D), a plate (2D), and a full plate (3D). A simple neural network model was used to predict material properties, which were then inputted into the finite difference solver to predict the displacement field. The method performed well for the 1D and 2D models, but the results for the 3D model were less satisfactory. Despite producing accurate results, this approach proved computationally intensive due to the integration of multiple finite difference schemes.

**Figure 13:**
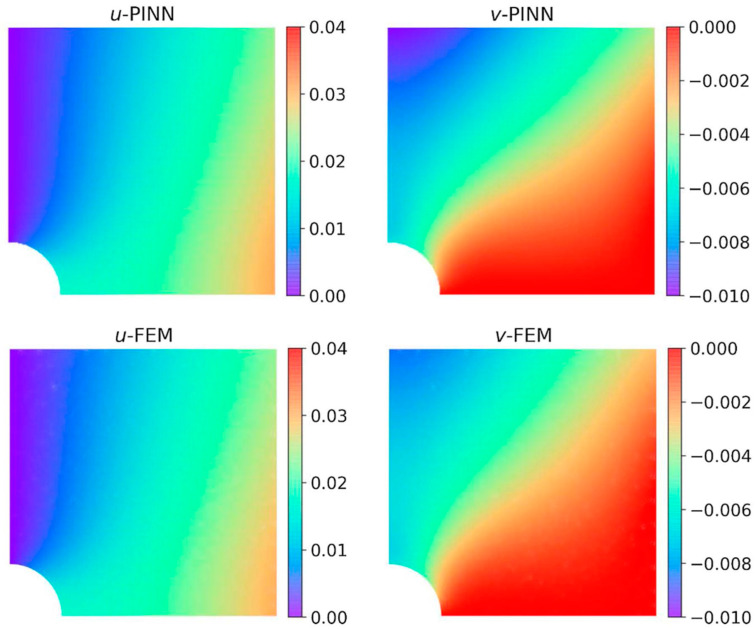
Comparison of the obtained displacement fields (PINN has a 6 × 60 net). Adapted from [146].

**Figure 14:**
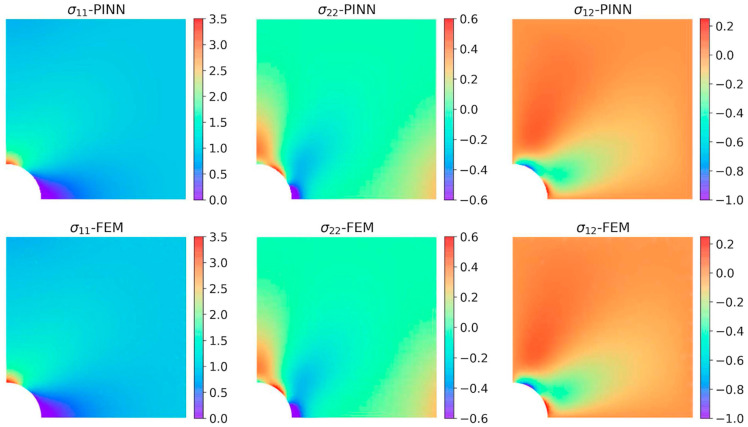
Comparison of the obtained stress fields (PINN has a 6 × 60 net). Adapted from [146].

The following year, Rao et al. [146] introduced an innovative approach for solving elastodynamic problems using a PINN model inspired by the Finite Element Method (FEM). This method was tested on a defective plate subjected to periodic uniaxial tension. To address the issues associated with soft constraint methods, the approach employed a hard constraint methodology. Notably, no labeled data was used for training. Instead, three separate deep neural networks (DNNs) were trained in an integrated manner to predict displacement and stress fields. The results were compared with the ground truth obtained from FEM solutions, as shown in Figure 13 and Figure 14.

Acoustic scattering is a widely utilized technique in Structural Health Monitoring (SHM), providing valuable insights into the geometry and composition of structures [147]. Wang et al. [148] trained a PINN model using scattered acoustic wavefield data. While no significant modifications were made to the traditional PINN model, they suggested increasing the number of hidden layers and neurons to tackle more complex problems. To evaluate the model’s performance, the authors advanced through a series of progressively complex problems, including the homogeneous velocity model, reflection model, layer model, single damage model, and multiple damage model. However, the model’s performance did not exceed that of traditional physics-based methods, primarily due to its smaller dataset and fewer theoretical formulations compared to conventional approaches.

Zargar and Yuan [86] trained a PINN model with sparse sensor data to reconstruct the 2D scattered Lamb wavefield for damage diagnosis. In addition to damage detection, the study also focused on identifying the impact location and reconstructing the impact force time history using a pre-trained CNN-LSTM model derived from the wavefield generated by the PINN. Furthermore, the study examined how environmental and operational conditions affected guided wave propagation.

**Figure 15:**
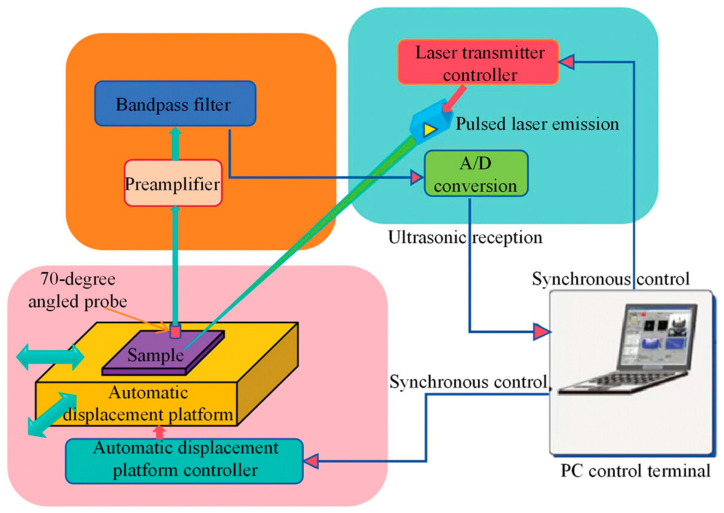
Laser ultrasonic testing system. Adapted from [149].

Next, Li et al. [149] used incomplete Laser Ultrasonic (LU) experimental data to train a PINN model for reconstructing a full 2D wavefield. Unlike earlier studies that relied on simulated data from numerical methods, this work was a pioneering effort to train a PINN model using experimental data. Figure 15 illustrates the experimental setup used for data collection.

In a more recent study, Chen et al. [150] introduced a variation of the PINN called two-step scaled PINN (TSS-PINN) to predict elastic modulus distribution along hull ribs, using simulated 1D elastic wave data to train the model.

In conclusion, the use of PINNs in wave equation-based SHM applications is still in its infancy, with most studies relying on simulated data generated from traditional numerical methods. However, ongoing research shows promising advancements, and the subsequent sections will review recent studies, showcasing the potential of PINNs in wave equation-based engineering applications and providing guidance for future research directions.

### 4.4. Identification of Material Properties

The study of material properties and their behavior is crucial for engineering design, failure analysis, and assessment of structural integrity [151,152]. Wave-based techniques, such as Surface Acoustic Wave (SAW) sensing, Scanning Acoustic Microscopy (SAM), and Line Focus Acoustic Microscopy (LFAM), are well-established methods for characterizing material properties [153,154,155]. Despite being in its early stages, research has begun exploring the potential of combining PINNs with traditional wave-based methods for material characterization. This section reviews studies that combine the traditional wave-based techniques with the PINN model to advance the characterization of material properties.

In 2021, Shukla et al. [156] first proposed a PINN model to compute the elastic stiffness (c_11_, c_12_, c_44_) of polycrystalline nickel. Here, c_11_ represents the linear combination of rigidity and compressibility. c_12 and_ c_44_ represent the rigidity of the material. The researchers employed separate PINN models for different components. For predicting the c_44_, two separate DNNs were used to perform forward and inverse problems. While predicting the c_44_, temporal coordinates were omitted_._ Similarly, when computing the c_12_ and c_11_, three separate DNNs were incorporated into the proposed PINN model. Both numerical and experimental data were used to train the model. Additionally, PCA was applied to filter high-frequency and low-energy data. For non-linear mapping, the self-adaptive activation function was utilized to achieve better convergence.

In another study, Lee and Popovics [157] tackled the inverse problem of characterization of material properties by predicting the variation of wave velocities along the length of two cylindrical rods made of two different materials. Only 20% of the experimental data was used for training. Rather than normalizing the data in the preprocessing stage, the researchers incorporated a normalization layer directly into the model architecture, preserving the physical significance of the input data while ensuring effective normalization.

Rathod and Ramuhalli [158] also used the PINN model to approximate material properties. The authors trained the vanilla PINN model with analytical solutions. However, the proposed model works better for constant or similar velocities, but it struggles to capture the abrupt changes in material properties. Wu et al. [159] studied the longitudinal and lateral vibration of a beam using 1D wave equation. The study involves determining the material properties of the beam by solving inverse problems through PINN. To solve the inverse problem, the authors experimented with both hard and soft constraint approaches. For this specific problem, the soft constraint approach offered better accuracy. Data sampling strategies for different cases were explored under the scope of this study. According to the analysis, with the increasing number of observational points, the relative error of the model is reduced by the order of magnitude.

**Figure 16:**
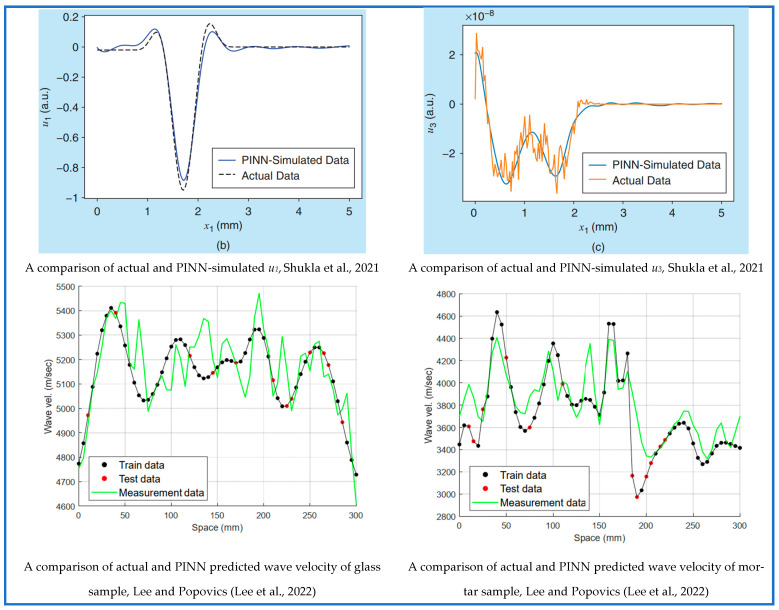
Performance comparison of PINN with established physics-based methods in material properties identification [156,157].

**Figure 17:**
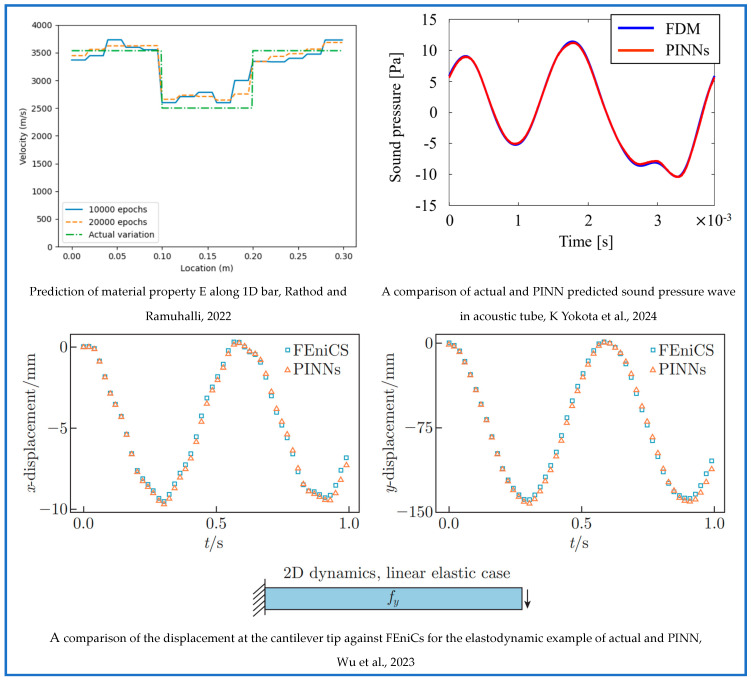
Performance comparison of PINN with established physics-based methods in material properties identification [158,159,160].

On the other hand, Yokota et al. [160] identified the energy loss parameter (G, R) of an acoustic tube by analyzing the sound waves using PINN. Here, G represents the energy loss due to heat conduction, and R represents the energy loss due to viscosity. For forward analysis, two separate DNNs were used. The first one takes the spatial–temporal coordinates as the input parameter and predicts the volume velocity and pressure field as the output. In the second one, only temporal coordinates were given as input to predict the volume velocity at the maximum length of the tube, ensuring the radiation boundary condition. In the loss term, the authors added two new loss terms named periodicity loss and coupling loss. The periodicity loss term confirms the steady state solution, and the coupling loss ensures that the model can catch the relationship between the sound wave pressure and volume velocity. As R has a negligible impact on the pressure wave, the proposed model failed to predict R, but it was successful in identifying the G. Compared to the studies in the SHM sector, researchers in this field have utilized both analytical and experimental data besides the computational one to train PINN models. Figure 16 and Figure 17 show the robustness of PINN models from different studies in the case of material properties identification.

### 4.5. Seismic Imaging

Since the development of the concept of seismic imaging in the 1920s, it has played a crucial role in exploring the earth’s subsurface, fault zone characterization and discovering energy resources. Over the years, this field has continuously improved in data acquisition, processing, and interpretation [161]. Full-waveform inversion, depth imaging, and travel time tomography are some established approaches utilized in seismic imaging/inversion [162].

In recent years, researchers have been utilizing the data derived from these traditional techniques to train the PINN model for computational ease and more accuracy. Notably, in 2019, Xu et al. [163] trained a PINN model using the analytical solution of the acoustic wave to predict velocity parameters for the first time. Simulation data from the Full Waveform Inversion (FWI) method was used to validate the outcome of the trained PINN model. In the subsequent years, there has been a substantial increase in the volume of work employing PINNs in this field, outpacing other fields in a relatively short period. In 2020, Karimpouli et al. [164] proposed a PINN model for elastic wave modeling in a 1D isotropic homogeneous medium. Kumar et al. [165] utilized the vanilla PINN model for subsurface velocity profile prediction in the 2D medium.

Waheed et al. [166] solved the acoustic qP eikonal wave equation, which describes the travel time of wavefronts in a medium based on the wave velocity for a 2D anisotropic medium. Unlike the other works, the authors solved this equation to predict the travel time from the source location. The trained model even works better for different source locations. This model dramatically reduces the computational time and effort needed to model the eikonal equation for an anisotropic medium. In another study, Smith et al. [167] presented an innovative application of PINNs for probabilistic earthquake hypocenter inversion. By training a PINN (EikoNet) to solve the eikonal equation, the authors effectively modeled the seismic wave travel times in a 3D velocity volume. The integration with Stein Variational Inference (SVI) allows for probabilistic hypocenter inversion, providing a robust and scalable solution for earthquake localization. Chen et al. [168] developed pinnET for solving the eikonal equation to predict the travel time and phase velocities of the Rayleigh wave through the medium. Unlike the eikonal equation solver proposed by Waheed et al. [166], Chen et al. used two NN models for two outputs. Arctan was utilized in this study instead of the regular tanh activation function. The solution of the pinnET was validated with other traditional surface wave tomography, eikonal tomography solutions, and ambient noise surface wave tomography results, which proves the success of the proposed pinnET model.

**Figure 18:**
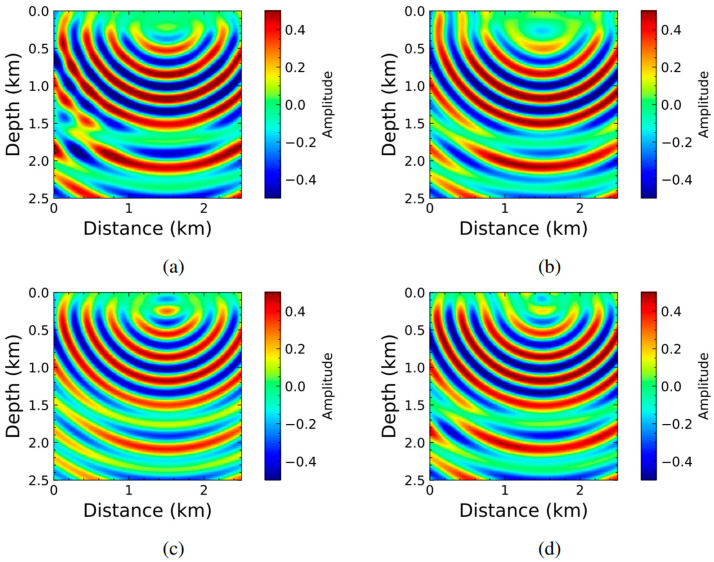
The real parts of scattered wavefield solution for a source located at 1.5 km via (**a**) numerical methods, (**b**) PINN using tanh without PE, (**c**) PINN using sine without PE, (**d**) PINN using sine with PE. Adapted from [169].

Song and Alkhalifah [169] solved the acoustic wave equation in the frequency domain for 2D and 3D homogeneous isotropic medium for reconstructing the wavefield. The scattered wavefield was simulated using vanilla PINN. Huang et al. [170] also addressed a similar problem with unlabeled data and significant modifications in the proposed PINN model for better convergence rate and accuracy. The authors solved the equation for the 2D problem. In this study, the input parameters were passed in the NN after performing the positional encoding (PE), transforming them into the higher dimensional space. This technique helped to alleviate the low-frequency bias of DNN. Also, instead of using traditional activation functions, the sine activation function was utilized. A comparative result among the three proposed approaches and the numerical methods for a single case (source located at 1.5 km) have been shown in Figure 18. In the first model, the researchers used the tanh activation function with PE, and in the second one, they used the sine activation function without PE. Lastly, PE and sine activation functions were utilized altogether, which showed lower errors than the other two models. However, the researchers were concerned about the chance of overfitting, and it was suggested that the second approach be used to address a similar problem.

In a study by Behesht et al. [171], a major modification to the vanilla PINN was introduced in the case of injecting the physical laws. The authors trained the PINN model with two early snapshot data, which inform the model about the initial conditions. In this study, 2D acoustic wave equation with absorbing boundary conditions (ABC) was solved. Though problems with absorbing boundary conditions seem notoriously challenging, the developed PINN model showed great performance without any labeled data. In the developed model, two different NN architectures were utilized for forward and inverse problems. For the forward problem, the NN outputs the wavefield potential. The displacement field is then simulated using automatic differentiation from deep learning libraries [50]. The second NN architecture provides the acoustic wave speed as the output parameter, similar to the other inverse problems. A total of five different cases were shown where the authors addressed different types of inhomogeneity and different kinds of sources and frequency. While the proposed model was successful in each of these cases, it faced challenges when applied to complex models such as the Marmousi model. In another study from Song et al. [172], the Helmholtz equation was solved using the PINN model with an adaptive sine activation function. The model was tested for different cases: isotropic homogeneous model with PML, isotropic Marmousi model, VTI (Vertical Transverse Isotropy) anomaly model, TTI (Tilted Transverse Isotropy) homogeneous model, and TTI model with irregular topography.

Zhang et al. [173] utilized PINN for seismic inversion using first-order acoustic wave equations. The trained model had been applied to estimate subsurface velocity and density fields of two-layered media, complex three-layered media, and salt body media. A key feature of this work is the use of well-logging data alongside partially observed seismogram surface data to enhance model accuracy. Another specialty is that the model does not require ABC to avoid the issues of the reflected wave of the boundary. Ding et al. [174] trained the Self-Adaptive PINN (SA-PINN) [49] using data from the spectral element method (SEM) to model 1D and 2D SH wave in infinite and semi-infinite domains. The predictability of the model was tested in complex topography, such as arc-shaped canyon and hill topography. To improve accuracy and convergence as well as to avoid temporal causality, the authors incorporated the sequential learning approach with the model. Sethi et al. [129] solved the acoustic wave equation for a heterogeneous medium using PINN. To address the spectral bias issues of the neural network model, a Fourier neural network was used in place of the traditional PINN. This approach also ameliorates the convergence rate of the model. P Ren et al. [175] proposed SeismicNet for modeling elastic wave equations in homogeneous and heterogeneous semi-infinite 2D domain. In this research work, the authors adopted approaches like parametric loading, truncation of domain using ABC and sequential training via time domain decomposition for improving accuracy. Also, the automatic differentiation facility was utilized to transform the 2nd order PDE into the 1st order PDE. Three different DNNs were used to approximate displacement, velocity, and stress fields to lessen the computational burden. In summary, this sector has seen a comparatively higher number of successful PINN applications, serving as a key inspiration for researchers from other fields. Given the depth of research in this area, this section demands a standalone review paper. Thus, further detailed discussions are beyond the scope of this section.

### 4.6. Medical Imaging

In 2021, Liu and Almekkawy [176] used PINN as an alternative approach to Ultrasound Computed Tomography (USCT) to solve the inverse problem of reconstructing tissue properties. While constructing the PINN model, the authors preferred using separate DNNs for forward and inverse problems. The developed model accurately estimated the speed of sound (SoS) of a 2D tissue phantom. The results demonstrate that PINNs can effectively handle the complex task of SoS reconstruction with an acceptable error margin. Jin et al. [177] proposed PINN for modeling 3D shear wave propagation in incompressible, transversely isotropic (ITI) materials, like skeletal muscle. The study covered the forward problem only. The proposed model was successful in simulating the displacement and pressure field based on the given spatial-temporal coordinates as input parameters. Though the proposed PINN model gives a slightly deviated result compared to the FEM solution, it takes almost 1/6 computational time to perform the same simulation.

**Figure 19:**
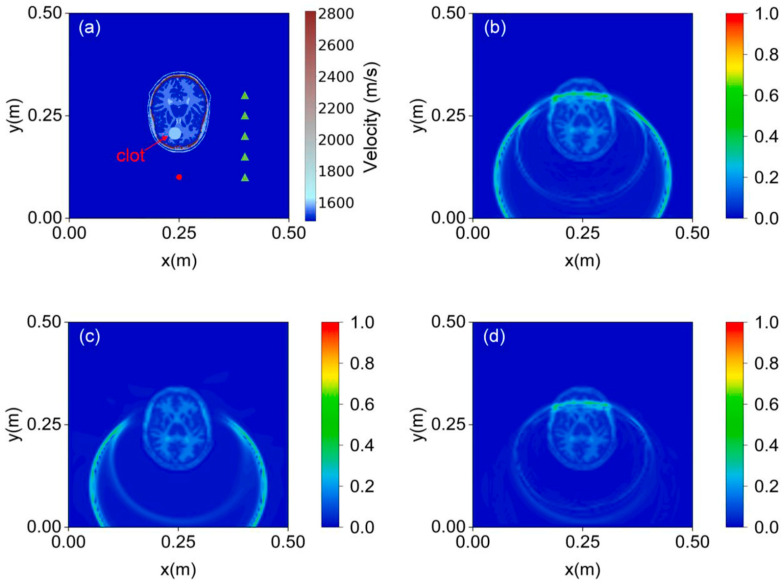
Comparison of the PINN’s wavefield prediction to the reference solution using the 2D brain model with a clot. (**a**) 2D brain model with a clot (the green triangles represent the receiving sensors); (**b**) the reference solution obtained by the SEM; (**c**) the prediction of PINN; (**d**) the difference between (**b**,**c**). Adapted from [178].

In a study by Wang et al. [178], the traditional PINN model was trained using two early-time snapshots of wavefield data generated by the spectral-element method (SEM) to predict the propagation of ultrasound waves through the skull. The authors examined the capability of the proposed model, implementing it into three different cases: homogeneous model, inhomogeneous model, and 2D brain model. In three of the cases, the model performed satisfactorily, though it faced difficulties while simulating reflected waves in lower amplitudes in 2D brain model. Also, it struggled to catch the high-velocity contrast. Figure 19 depicts the comparative result between the proposed PINN model and the wavefield simulated using SEM for the 2D brain model. In a recent study by Yin et al. [179], SWENet was proposed for estimating the elastic properties of inhomogeneous soft material. The researchers used ample amounts of data to train the model from three different sources in addition to the incorporation of the physical laws through regularization terms. Two separate DNNs were used for forward and inverse problems. In this study, the transfer learning approach was proposed for faster training using different datasets where users can utilize the weights and bias parameters from the previous training step. The effect of the noisy data and multi-source data on the proposed model was also covered in this study.

**Table 2:** A selective list of studies leveraging PINN for modeling wave propagation for addressing different engineering problems.

Area of Application	Authors	Year	Key Objectives	Type of Wave	Dimension	Type of Medium
*Structural Health Monitoring*	Shukla et al. [144]	2020	Identification and characterization of surface-breaking crack	Acoustic wave	2D	Homogeneous Heterogeneous
	Rao et al. [146]	2021	Solving computational elastodynamic problem	Elastic wave	2D	Homogeneous
	Ghellero [145]	2022	Damage localization	Lamb wave	1D, 2D, 3D	Inhomogeneous, Heterogeneous
	Wang et al. [148]	2023	Damage detection	Acoustic wave	2D	Homogeneous Inhomogeneous
	Zargar, and Yuan [86]	2024	Damage diagnosis and impact location study of an aluminum plate	Lamb wave	2D	Homogeneous
	Li et al. [149]	2024	Reconstructing incomplete ultrasonic wavefield	Acoustic wave	2D	Homogeneous
	Chen et al. [150]	2025	Solving inverse problem to estimate elastic modulus of hull ribs	Elastic wave	1D	Inhomogeneous
*Identification of Material Properties*	Shukla et al. [156]	2021	Quantification of the microstructural properties of polycrystalline nickel	Elastic wave	2D	Isotropic
	Lee and Popovics [157]	2022	Characterization of in-place material properties	Elastic wave	1D	Inhomogeneous
	Rathod and Ramuhalli [158]	2022	Estimation of the material properties	Standing waves	1D	Homogeneous Inhomogeneous
	Wu et al. [159]	2023	Identification of unknown material parameters	Elastic wave	1D	Homogeneous
	K Yokota et al. [160]	2024	Identification of loss parameter	Acoustic wave	1D	Inhomogeneous
*Seismic Imaging*	Xu et al. [163]	2019	Velocity inversion	Acoustic Wave	2D	Not mentioned
	Karimpouli et al. [164]	2020	Velocity inversion	Elastic Wave	1D	Isotropic, Homogeneous
	Kumar et al. [165]	2020	Subsurface velocity profile prediction	Seismic Wave	2D	Not mentioned
	Waheed et al. [166]	2020	Travel time computation	Acoustic qP Wave	2D	Anisotropic
	Song and Alkhalifah [169]	2021	Wavefield reconstruction	Acoustic Wave	2D 3D	Isotropic, Homogeneous
	Huang et al. [170]	2021	Solving scattered wavefield	Acoustic Wave	2D	Not mentioned
	Behesht et al. [171]	2022	Seismic Inversion	Acoustic Wave	2D	Heterogeneous
	Song et al. [172]	2022	Illumination of subsurface	Acoustic	3D	Anisotropic
	Smith et al. [167]	2022	Probabilistic earthquake hypocenter inversion	P—wave and S-wave	3D	Heterogeneous
	Chen et al. [168]	2023	Eikonal Tomography	Rayleigh Wave	2D	Not mentioned
	Zhang et al. [173]	2023	Seismic Inversion	Acoustic wave	2D	Heterogeneous
	Ding et al. [174]	2023	Modeling Seismic wave	SH wave	1D 2D	Homogeneous
	Sethi et al. [129]	2023	Modeling Acoustic Wavefield	Acoustic wave	2D	Heterogeneous
	Ren et al. [175]	2024	Seismic wave modeling	Elastic wave	2D	Homogeneous Heterogeneous
*Medical Imaging*	Liu and Almekkawy [176]	2021	Reconstruction of tissue properties	Acoustic wave	2D	Heterogeneous
	Jin et al. [177]	2022	Studying incompressible transversely isotropic tissues	Elastic wave	3D	Transversely Isotropic
	Wang et al. [178]	2023	Simulating ultrasound wave propagating through skull	Acoustic	2D	Homogeneous Inhomogeneous
	Yin et al. [179]	2023	Measuring elastic properties of soft material	Elastic	2D	Heterogeneous

## 5. Conclusions

It is highly evident from the above presentation that SciML models have made significant progress within a short span of time. There is no doubt that certain SciML models possess remarkable potential to emerge as highly effective PDE solvers. However, the question still remains: can they truly surpass the gold standard of traditional numerical methods? PINN, being a relatively new approach among the four discussed SciML, has attracted significant research interest due to its efficacy. Section 4 reflects on some key advantages of PINNs: low memory requirements, no limitations on the shape of the solutions, flexibility for different types of initial and boundary conditions, meshless formalism, free of numerical dispersion artifacts, no need for calculating impedance matrix, efficient for irregular topography, advantages of easier gradient calculation, inverse problem optimization using GPUs easily, quicker computation time, forward and inverse problem in the same optimization problem set, high prediction speed, and interpolate and extrapolate facility.

There are several promising avenues for future research to enhance the performance of PINNs in wave propagation modeling. One key area involves addressing the challenges posed by non-convex solutions, as gradient-based optimizers often struggle with such problems. Several studies have highlighted this limitation, emphasizing the need for further investigation to develop robust optimization techniques. Although PINNs offer significant speedup in simulations after successful training, the models require substantial time for training. Additionally, their problem-specific nature presents a challenge, as the models must be retrained whenever physical constraints change. This limitation underscores the importance of focusing on generalization to improve usability. This issue can be solved using pretrained models leveraging transfer learning paradigms. The current PINN framework primarily relies on simple forward propagation within feedforward neural networks (FFNNs). However, it does not yet incorporate more advanced architectures such as convolutional neural networks (CNNs), recurrent neural networks (RNNs), graph neural networks (GNNs), or generative adversarial networks (GANs). Integrating these architectures could significantly enhance the flexibility and capability of PINNs. Another major problem with PINN is that there are no guidelines yet to optimize the model. Many researchers have questioned the reliability of the outcomes of these models as these can be seen as a black box and there are not many resources that can measure the explainability of NN-based models.

This study revealed that geophysics researchers are notably more active in this field compared to other sectors, indicating significant opportunities for experts in the SHM field to explore this valuable integration of scientific computing and deep learning. Despite being in the nascent stage of development, the field of PINN has progressed far beyond expectations. The research works highlight the vast potential of PINN in scientific computing, indicating significant opportunities for future breakthroughs.

## Data Availability

Not applicable.
